# Adipose MDM2 regulates systemic insulin sensitivity

**DOI:** 10.1038/s41598-021-01240-3

**Published:** 2021-11-08

**Authors:** Philip Hallenborg, Benjamin Anderschou Holbech Jensen, Even Fjære, Rasmus Koefoed Petersen, Mohammed-Samir Belmaâti, Sarah Søndergård Rasmussen, Jon Petur Gunnarsson, Pernille Lauritzen, Kenneth King Yip Cheng, Martin Hermansson, Si Brask Sonne, Christer S. Ejsing, Aimin Xu, Irina Kratchmarova, Marcus Krüger, Lise Madsen, Karsten Kristiansen, Blagoy Blagoev

**Affiliations:** 1 Department of Biochemistry and Molecular Biology, University of Southern Denmark, 5230 Odense M, Denmark; 2 Laboratory of Genomics and Molecular Biomedicine, Department of Biology, University of Copenhagen, 2100 Copenhagen Ø, Denmark; 3 Institute of Marine Research, 5817 Bergen, Norway; 4 Department of Health Technology and Informatics, Hong Kong Polytechnic University, Hong Kong, Hong Kong; 5 Cell Biology and Biophysics Unit, European Molecular Biology Laboratory, 69117 Heidelberg, Germany; 6 State Key Laboratory of Pharmaceutical Biotechnology, University of Hong Kong, Hong Kong, Hong Kong; 7 Department of Medicine, University of Hong Kong, Hong Kong, Hong Kong; 8 Department of Pharmacology and Pharmacy, University of Hong Kong, Hong Kong, Hong Kong; 9 Excellence Cluster on Cellular Stress Responses in Aging-Associated Diseases (CECAD) and Center for Molecular Medicine Cologne (CMMC), University of Cologne, 50931 Cologne, Germany; 10 Institute for Metagenomics, BGI-Shenzhen, Shenzhen, 518083 People’s Republic of China

**Keywords:** Biochemistry, Cell biology, Molecular biology, Physiology, Diseases

## Abstract

The intimate association between obesity and type II diabetes urges for a deeper understanding of adipocyte function. We and others have previously delineated a role for the tumor suppressor p53 in adipocyte biology. Here, we show that mice haploinsufficient for MDM2, a key regulator of p53, in their adipose stores suffer from overt obesity, glucose intolerance, and hepatic steatosis. These mice had decreased levels of circulating palmitoleic acid [non-esterified fatty acid (NEFA) 16:1] concomitant with impaired visceral adipose tissue expression of *Scd1* and *Ffar4*. A similar decrease in *Scd* and *Ffar4* expression was found in in vitro differentiated adipocytes with perturbed MDM2 expression. Lowered MDM2 levels led to nuclear exclusion of the transcriptional cofactors, MORC2 and LIPIN1, and thereby possibly hampered adipocyte function by antagonizing LIPIN1-mediated PPARγ coactivation. Collectively, these data argue for a hitherto unknown interplay between MDM2 and MORC2/LIPIN1 involved in balancing adipocyte function.

## Introduction

Dysfunctional adipocytes with impaired ability to expand and diminished ability for uptake of glucose are linked to ectopic lipid accumulation in muscle and liver. Hepatic buildup of neutral lipids (i.e., triacylglycerol and cholesteryl ester) eventually results in the development of non-alcoholic fatty liver disease (NAFLD) closely associated with type 2 diabetes (T2D). Perturbed secretion of signaling molecules from adipocytes of both protein and lipid origin, commonly referred to as adipokines, also contributes to systemic metabolic malfunctioning^1,2^.

The expression of adipokines is tightly regulated by a myriad of transcription factors. Obesity disturbs the balance between these transcriptional regulators. Peroxisome proliferator-activated receptor γ (PPARγ) and CCAAT/Enhancer-binding protein α (C/EBPα) are the two main regulators of adipocyte development and function. Anomalies in their expression, recruitment of other transcriptional regulators as well as post translational modification status all impact on adipocyte function^3,4^.

Other transcription factors, including the classic tumor suppressor p53, are known to also play pivotal roles in the regulation of adipocyte biology, and accumulating evidence points to a decisive role for p53 in balancing metabolism^5^. A number of reports point to a profound effect of p53 activation on adipocyte homeostasis^6–10^. More specifically, when activated by nutritional stress such as excessive caloric intake, p53 appears to act as a repressor of adipose insulin sensitivity and energy expenditure^7,10^. Emerging data also emphasize p53 as the mediator of age-induced fat loss^6,11^.

Given its central role in cellular homeostasis, the activity and expression of p53 are kept in check by various mechanisms. One of the best described relates to the function of the ubiquitin ligase Murine Double Minute 2 (MDM2). By attaching ubiquitin moieties, MDM2 targets p53 for nuclear export and proteasomal degradation. Despite their tight association, it is by now well appreciated that MDM2 also engages in p53-independent pathways^12^.

Little is known about the involvement of MDM2 in adipocyte biology. We have previously demonstrated an involvement of MDM2 in the very early steps of adipocyte differentiation^13,14^. Of note, this role was likely independent of the ubiquitin ligase activity of MDM2 as ectopic expression of the N-terminal half of MDM2, devoid of ubiquitin ligase domain, was able to restore adipogenesis in fibroblasts lacking *Mdm2*^13^.

Recently, an age-associated decline in adipose MDM2 was shown to cause unrestricted p53-activation leading to lipodystrophy^11^. These data suggest a pivotal role of MDM2 in adipocyte development and function. Here, we substantiate this notion further by showing that haploinsufficiency of MDM2 in adipose tissue leads to marked increase in adipose tissue mass, glucose intolerance and hepatic steatosis in young mice. Mechanistically, reduced MDM2 level attenuates expression of the G-protein coupled receptor GPR120 (encoded by the *Ffar4* gene) at least in part independent from p53. MDM2 regulated the cellular localization of the transcription cofactors MORC2 and LIPIN1, of which the latter is a known PPARγ coactivator^15^. Taken together, our results point to a role for MDM2 in orchestrating adipose homeostasis at least in part through a novel interplay with the transcriptional cofactors MORC2 and LIPIN1.

## Results

### Decreased levels of adipose *Mdm2* leads to impaired glucose homeostasis

Because p53 plays a pivotal role in the regulation of metabolism and adipose function, we explored the importance of its key regulator, MDM2, in adipocyte biology. We have previously reported a requirement for this ubiquitin ligase in adipocyte differentiation^13,14^. Since several genes known to affect adipogenesis are also essential for adipocyte function, we set out to explore the role of MDM2 in mature adipocytes. While we did not find changes in *Mdm2* levels during adipose differentiation in cultured adipocytes (Supplementary Fig. S1a), in agreement with previous finding^16^, we found augmented expression in the visceral epididymal adipose tissue (epiWAT) compared to subcutaneous inguinal white (ingWAT) and intrascapular brown (intBAT) adipose tissues (Supplementary Fig. S1b). Given that the expression of several genes involved in adipose tissue homeostasis is known to change in response to metabolic challenges and obesity development, we examined the levels of *Mdm2* mRNA in the white adipose tissue (WAT) of diet-induced obese mice (Supplementary Fig. S1c) and genetically obese, *ob/ob*, mice (Supplementary Fig. S1d). In both instances, we observed a two-fold increase in the level of *Mdm2* expression compared to lean mice, while no increase was observed in liver and muscle.

To examine if the augmented *Mdm2* expression in WAT in the obese state had physiological relevance, we generated mice lacking one allele of *Mdm2* in their adipose tissue, hereafter referred to as *Mdm2*^Adi+/−^ mice. In short, mice bearing loxP sites in the *Mdm2* gene were crossed with mice expressing the Cre recombinase under control of the *Fabp4* promoter^17,18^. Although both loxP and Cre-expressing mice were on a C57BL/6J background, the mice were bred more than 10 generations to avoid possible interference from substrain differences. As MDM2 is difficult to detect in vivo, we enriched for MDM2 in lysates from epiWAT using immunoprecipitation with the one monoclonal antibody (SMP14) prior to western blotting with another monoclonal antibody (4B2). As expected, the level of MDM2 protein was decreased in the adipose tissue of *Mdm2*^*Adi*+*/−*^ mice (Supplementary Fig. S1e). The lowered expression of *Mdm2* was also confirmed at mRNA level in the ingWAT and intBAT depots (Supplementary Fig. S1f.). Furthermore, the reduction of *Mdm2* expression was confined to the adipocyte fraction (AF) of the adipose depot and not occurring in the stromal vascular fraction (SVF) (Supplementary Fig. S1g).

**Figure 1:**
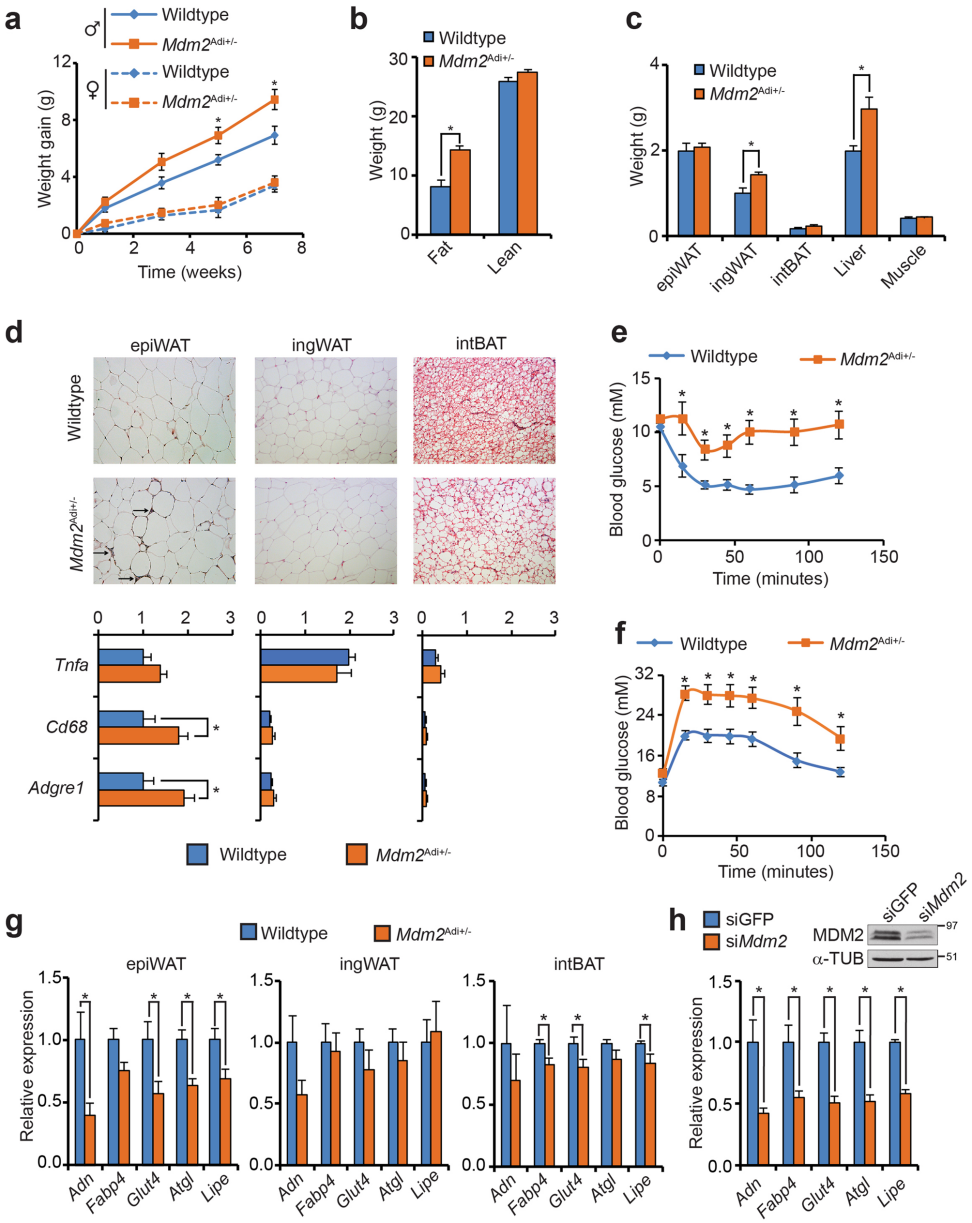
Male mice lacking one allele of *Mdm2* in their adipose exhibit accentuated fat accumulation and impaired insulin sensitivity when fed a high-fat diet. (**a**) Weight gain of male and female wildtype (N = 8 and 10, respectively) and *Mdm2*^Adi+/−^ mice (N = 7 and 9, respectively). Mice were 6 weeks of age at the onset of high-fat feeding. (**b**) Fat and lean mass of wildtype (N = 8) and *Mdm2*^Adi+/−^ (N = 7) male mice scored by MR scanning after 10 weeks of high-fat feeding. (**c**) Weight of isolated tissues after 15 weeks on high-fat diet. (**d**) Histological inspection (top) and macrophage marker gene expression (bottom) by real-time qPCR of epiWAT, ingWAT and intBAT from high-fat fed wildtype and *Mdm2*^Adi+/−^ mice after 15 weeks on high-fat diet. *Tnfa*, Tumor necrosis factor alpha; *Cd68*, Cluster of differentiation 68; *Adgre1*, Adhesion G protein-coupled receptor E1. (**e**) Insulin tolerance test after 13 weeks on high-fat diet. (**f**) Oral glucose tolerance test after 11 weeks on high-fat diet. (**g**, **h**) Real-time qPCR-based quantification of *Adn*, *Fabp4, Glut4, Atgl* and *Lipe* in (**g**) epiWAT, ingWAT and intBAT of high-fat fed wildtype and *Mdm2*^Adi+/−^ mice after 15 weeks on high-fat diet or in (**h**) 3T3-L1 adipocytes with knockdown of *Mdm2*. *Adn*, Adipsin; *Fabp4*, Fatty acid binding protein 4; *Glut4*, glucose transporter 4; *Atgl*, adipose triglyceride lipase; *Lipe*, hormone sentitive lipase. (**h**) Insert, western blot analysis of MDM2 and α-Tubulin. For **a**, **e**, and **f**, significance was tested using two-way ANOVA with Bonferroni-correction, * = *p*-value < 0.05. For **b**, **c**, **d**, **g**, and **h**, significance was tested using Student’s *t*-test, * = *p*-value < 0.05.

We challenged young *Mdm2*^Adi+/−^mice with a high-fat diet. Male *Mdm2*^Adi+/−^ mice gained more weight compared to wildtype littermates whereas female *Mdm2*^Adi+/−^ mice where indistinguishable from their wildtype littermates (Fig. 1a). The more dramatic development in obesity in male mice is well in line with literature^19^ and the cause for using male C57/BL6J mice for metabolic studies. We therefore chose to focus on the male cohort in the subsequent metabolic characterization.

The difference between male wildtype and *Mdm2*^Adi+/−^ mice was already evident after five weeks on high-fat diet (Fig. 1a) despite similar food intake and energy expenditure (Supplementary Fig. S1h, i). The elevated weight was due to increased fat mass and not lean mass (Fig. 1b). Although the ingWAT and intBAT as well as the liver were enlarged in *Mdm2*^Adi+/−^ mice, the size of the epiWAT was indistinguishable from wildtype mice (Fig. 1c). Histological inspection revealed a profound infiltration of the epiWAT by macrophages, which was confirmed by marker gene expression. In addition, we observed increased lipid accumulation in intBAT (Fig. 1d). In the two white adipose depots, the size of the adipocytes was only increased in ingWAT (Supplementary Fig. S1i). Hypertrophy without concomitant infiltration by immune cells is in accordance with the previously reported high adaptability of ingWAT^20^.

As overt obesity and inflammation of adipose stores are prognostic markers for diabetes development, we tested the glucose and insulin tolerance of the *Mdm2*^Adi+/−^ mice. Indeed, *Mdm2*^Adi+/−^ mice suffered from impaired glucose and insulin tolerance (Fig. 1e, f) pointing to a role of adipose MDM2 in supporting systemic insulin sensitivity. In this respect, it is worth noting that a microarray study hinted towards a lowered expression of *MDM2* in the visceral, but not subcutaneous adipose tissue of non-obese T2D patients compared to age- and BMI-matched healthy subjects (Supplementary Fig. S1k)^21,22^.

In line with the suggested impaired adipose function, the expression level of several genes essential for adipose function was significantly lowered in epiWAT of *Mdm2*^Adi+/−^ mice, but not in ingWAT and only marginally in intBAT (Fig. 1g) thereby mirroring the lowered *MDM2* expression observed only in the visceral depots of human diabetics. It is well-established that genes can be important for only one depot despite being expressed in all. A prototypical example is almost complete absence of white, but not brown and mammary gland fat tissues, in mice lacking the adipose master regulator C/EBPα^23^. It is plausible that the effect of lowered levels of MDM2 in *Mdm2*^Adi+/−^ mice was most severe in the epiWAT due the higher levels of *Mdm2* in this depot (Supplementary Fig. S1b). To assess if this perturbed gene expression was a cell autonomous phenomenon caused by the lowered MDM2 expression, we manipulated MDM2 levels in in vitro differentiated adipocytes. Knockdown or overexpression of *Mdm2* lowered or increased, respectively, the expression of the same set of genes (Fig. 1h, Supplementary Fig. S1l). Of note, *Mdm2*^Adi+/−^ mice fed a standard chow diet also exhibited enlargement of ingWAT and decreased insulin sensitivity (Supplementary Fig. S1m–o). Collectively, these data show that adipose MDM2 is necessary for proper adipose function in mice.

### ***Mdm2***^Adi+/−^ mice exhibit overt hepatic steatosis

**Figure 2:**
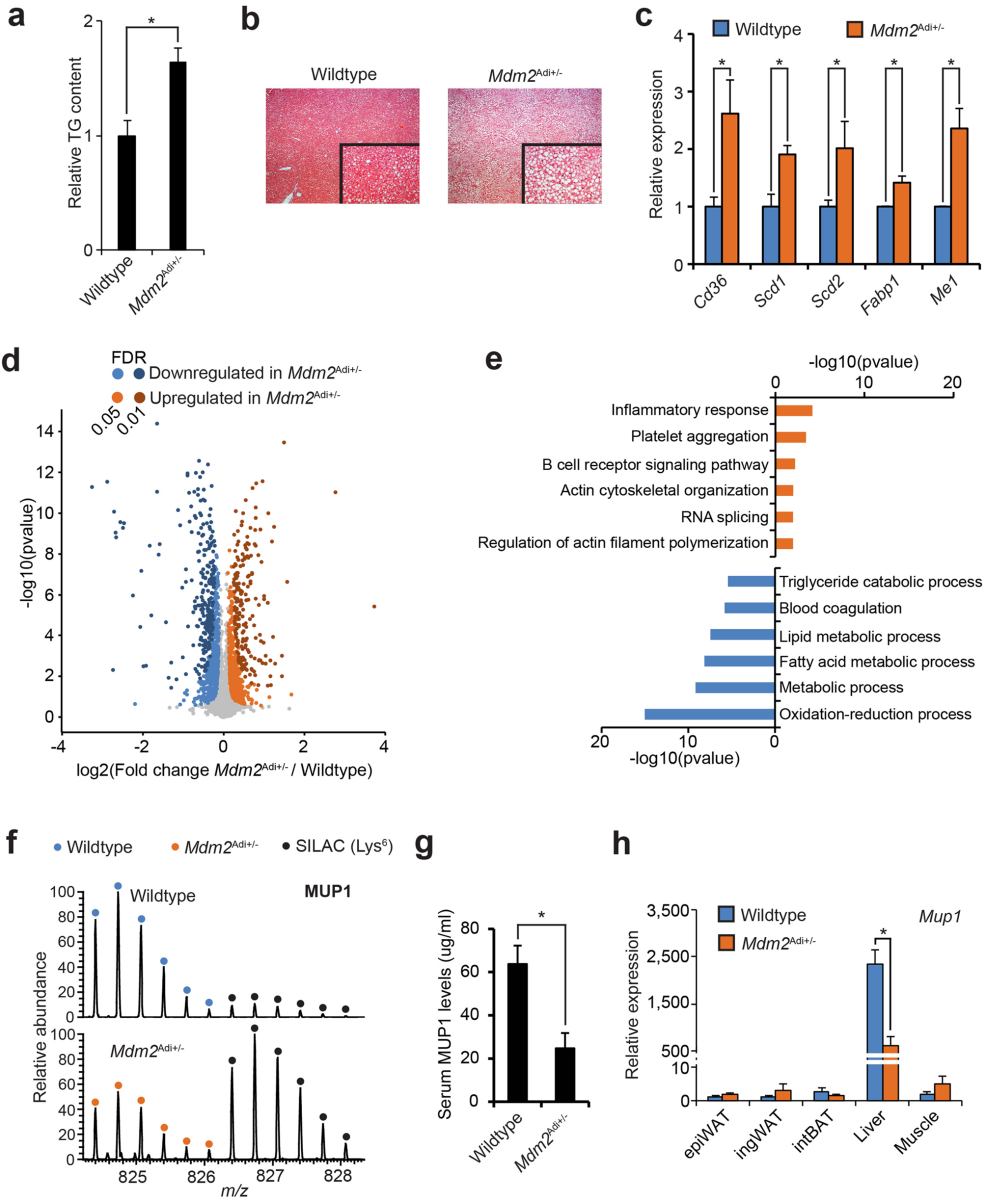
High-fat fed *Mdm2*^Adi+/−^ mice suffer from hepatic steatosis. (**a**, **c**, **d**, **g**, **h**) Male wildtype (N = 8) and *Mdm2*^Adi+/−^ (N = 7) mice after 15 weeks on high-fat diet. (**a**) Relative triglyceride content in livers normalized to weight. Triglycerides were extracted using isopropanol and quantified with an enzyme-based colorimetric assay. (**b**) Histological analysis of H&E-stained sections of livers. (**c**) mRNA levels of genes encoding lipid-handling proteins measured by real-time qPCR. *Cd36*, Cluster of differentiation 36; *Scd*, Stearoyl-CoA desaturase; *Fabp1*, Fatty acid-binding protein 1; *Me1*, malic enzyme 1. (**d**) Quantitative proteomic comparison of epiWAT from wildtype and *Mdm2*^Adi+/−^ mice fed a high fat diet for 15 weeks. Volcano plots of quantified proteins generated in Perseus^70^. FDR was set to either 0.05 or 0.01. s0 held at 0.1. (**e**) Enriched biological processes amongst the significantly up- or down-regulated proteins using DAVID 6.8^71,72^. (**f**) MS1 spectrum of a MUP1 (Major Urinary Protein 1) peptide (DGETFQLMGLYGREPDLSSDIK) from wildtype and *Mdm2*^Adi+/−^ mice. Depicted using Xcalibur. (**g**) ELISA-based quantification of serum levels of MUP1 in wildtype and *Mdm2*^Adi+/−^ mice after 15 weeks on high-fat diet. (**h**) mRNA levels of *Mup1* in adipose depots, liver, and muscle of high-fat fed wildtype and *Mdm2*^Adi+/−^ mice as scored by real-time qPCR. For **a**, **c**, **g** and **h**, significance was tested using Student’s *t*-test, * = *p*-value < 0.05. For **d**, *p*-value was calculated using Student’s *t*-test. For **e**, *p*-value was calculated using Fisher’s Exact test with Benjamini correction.

With overt obesity, where the storage capacity of the adipose tissues is exceeded, lipids are deposited systemically and most notably in the liver. The enlarged mass of livers in *Mdm2*^Adi+/−^ mice hinted that these mice suffered from hepatic steatosis (Fig. 1c). Indeed, both measurement of the relative TG levels and histological examination revealed a profound increase in hepatic lipid content in high-fat fed *Mdm2*^Adi+/−^ mice (Fig. 2a, b). Although we did not observe a significantly increased expression of genes involved in de novo lipogenesis or gluconeogenesis (Supplementary Fig. S2a, b), the livers of *Mdm2*^Adi+/−^ mice had augmented mRNA levels of enzymes crucial for lipid handling (Fig. 2c).

We next used a quantitative SILAC-based mass spectrometric approach to delineate proteomic differences at a global scale in epiWAT of wildtype and *Mdm2*^Adi+/−^ mice. We selected epiWAT, because we observed the largest effects in relation to gene expression in this depot. In short, lysates of epiWATs were mixed with lysates of adipose depots from SILAC-labeled mice^24,25^ (Supplementary Fig. S2c). We quantified 3,951 proteins of which 775 and 609 (FDR 0.05) were significantly downregulated or upregulated, respectively, in epiWAT of *Mdm2*^Adi+/−^ mice (Fig. 2d, Supplementary Table S1). GO-term enrichment analysis of biological processes revealed a prominent downregulation of proteins involved in lipid handling in *Mdm2*^Adi+/−^ mice (Fig. 2e). In agreement with the increased macrophage infiltration (Fig. 1d), we found an overrepresentation of proteins associated with the inflammatory response upregulated in *Mdm2*^Adi+/−^ mice (Fig. 2e). We also observed an enrichment of proteins associated with biological processes regulating actin cytoskeleton organization; a signature that was further emphasized when performing GO-term enrichment based on molecular functions (Supplementary Fig. S2d). The downregulated molecular functions were involved in regulation of the oxidative-reductive capacity. This was underscored by lower levels of almost all proteins of the mitochondrial complex I in the *Mdm2*^Adi+/−^ mice (Supplementary Fig. S2e).

Amongst the extremes in our dataset, we found a remarkable reduction in the level of the hepatokine MUP1 (Major Urinary Protein 1) in the epiWAT of *Mdm2*^Adi+/−^ mice (Fig. 2f, Supplementary Fig. S2f.). MUP1 has previously been shown to be important for maintaining both systemic insulin sensitivity and energy expenditure, and its expression is lowered in the context of hepatic steatosis^26–28^. Since MUP1 is selectively expressed in and secreted by the liver, its presence in the adipose tissue is of non-adipose origin, and rather reflects hepatic output. Indeed, *Mdm2*^Adi+/−^ mice had lower circulating levels of MUP1 and decreased expression of *Mup1* in their livers as confirmed by ELISA and qPCR, respectively (Fig. 2g, h) in agreement with the hepatic steatosis seen in these mice. Together with a general decrease in the levels of blood microparticles (Supplementary Fig. S2g), most of which are produced in the liver, our data point to a dysfunctional liver in *Mdm2*^Adi+/−^ mice, arguing for perturbed visceral fat-liver crosstalk in these mice.

### ***Mdm2***^Adi+/−^ mice display drastic changes in the levels of circulating palmitoleic acid

**Figure 3:**
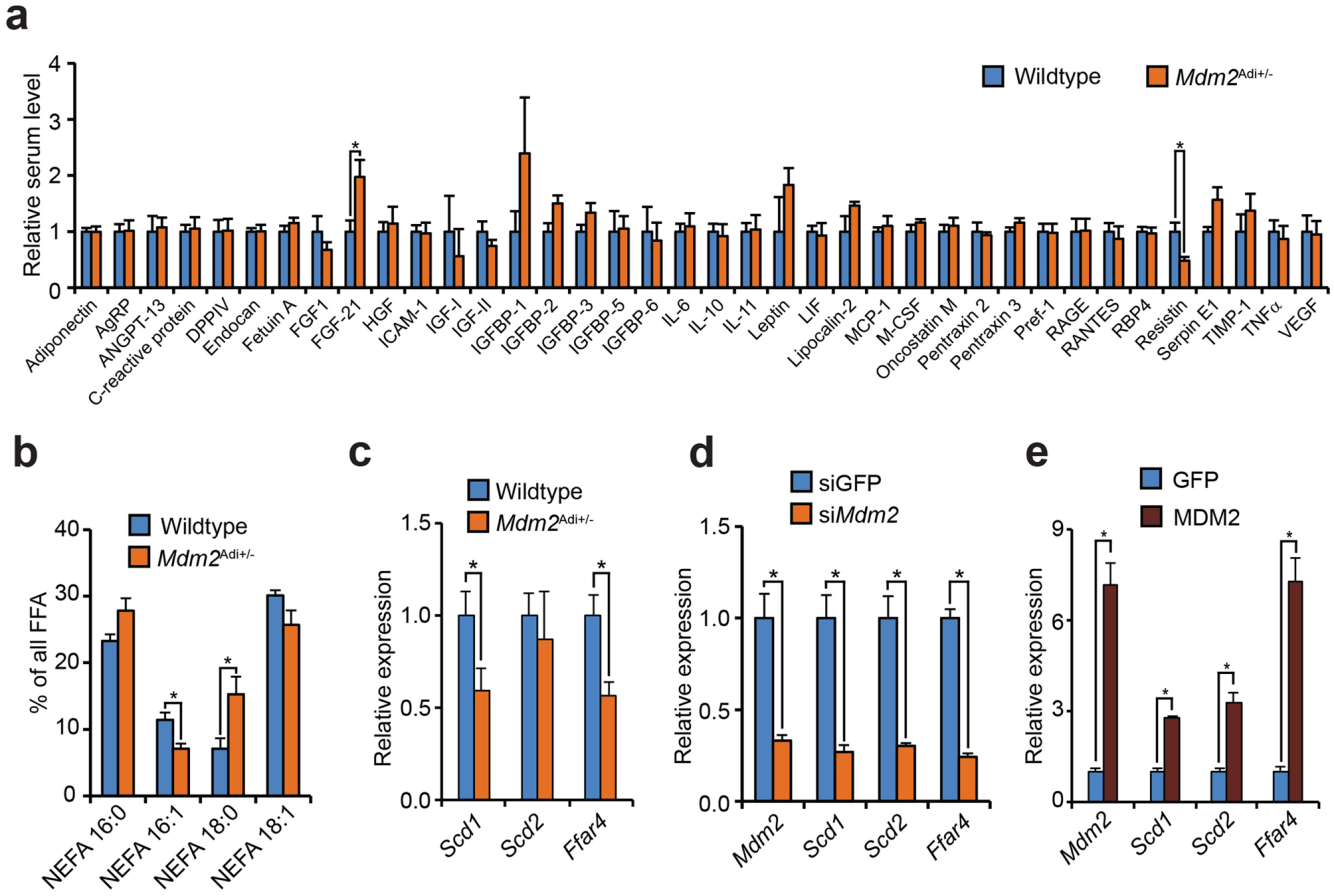
High-fat fed *Mdm2*^Adi+/−^ mice have disturbed adipokines secretion. (**a**, **b**) Serum from male wildtype (N = 4) and *Mdm2*^Adi+/−^ (N = 4) mice after 15 weeks on high-fat diet. (**a**) Relative circulating levels of 38 adipokines quantified with Adipokine Proteome Profiler Array. (**b**) Serum levels of NEFA 16:0 (free palmitic acid), NEFA 16:1 (palmitoleic acid), NEFA 18:0 (stearic acid) and NEFA 18:1 (oleic acid or vaccinic acid) quantified by mass spectrometry. (**c–e**) Real-time qPCR-based quantification of *Scd1*, *Scd2* and *Ffar4* in (**c**) epiWAT of wildtype (N = 8) and *Mdm2*^Adi+/−^ (N = 7) mice after 15 weeks on high-fat diet, in (**d**) 3T3-L1 adipocytes with knockdown of *Mdm2*, and in (**e**) 3T3-L1 adipocytes electroporated with plasmids expressing GFP or MDM2. *Ffar4*, Free fatty acid receptor 4. For all panels, significance was tested using Student’s *t*-test, * = *p*-value < 0.05.

Steatosis often develops during obesity when excess energy intake exceeds the capacity of adipose expansion. This leads to augmented circulating levels of TG and non-esterified fatty acids (NEFA). Curiously, if anything *Mdm2*^Adi+/−^ mice had decreased blood levels of total TG and NEFA (Supplementary Fig. S3a). Because adipocytes can regulate hepatic lipid deposition through endocrine signaling, we first measured the expression of protein adipokines reported to affect hepatic steatosis development^1^. The expression levels of *AdipoQ*, *Rbp4,* and *Retn* were lower in the epiWAT of *Mdm2*^Adi+/−^ mice than in wildtypes littermates (Supplementary Fig. S3b). Yet, as these decrements were restricted to the epiWAT, expression levels may not necessarily reflect the circulating levels. We therefore screened the serum for the presence of well-known protein adipokines. Of the 38 tested, only FGF21 and Resistin differed significantly between wildtype and *Mdm2*^Adi+/−^ mice (Fig. 3a). As the observed changes (increased FGF21 and lowered Resistin in *Mdm2*^Adi+/−^ mice) would be expected to increase insulin sensitivity^1,29^ we hypothesize that such alterations might represent a compensatory biological response to counteract decreased insulin sensitivity in *Mdm2*P^Adi+/−^P mice. Besides adipokines of protein origin, adipose stores are known to secrete several bioactive lipid molecules. Interestingly, mass spectrometric analysis of serum revealed a significant decrease in the circulating level of NEFA 16:1 (corresponding to palmitoleic acid) as well as augmented level of NEFA 18:0 (corresponding to stearic acid) (Fig. 3b) of which the former has been shown to be crucial for preserving systemic glucose homeostasis ^30^. Previous results have shown that the lipid sensor G-protein coupled receptor 120 (GPR120, encoded by *Ffar4*) is required for adipose NEFA 16:1 production by controlling *Scd1* expression^31^. The stearoyl-CoA desaturases (SCDs) are responsible for converting C16:0 and C18:0 into C16:1 and C18:1, respectively. Interestingly, the levels of not only *Ffar4* mRNA but also one of the two *Scd*s mRNAs reported to be expressed in adipose stores, *Scd1*, were lower in epiWAT of *Mdm2*^Adi+/−^ mice (Fig. 3c).

To again explore if this was a cell autonomous effect directly related to the lowered MDM2 level, we knocked down *Mdm2* mRNA in mature adipocytes in vitro. In agreement with the in vivo data, adipocytes with lowered levels of *Mdm2* mRNA had decreased levels of *Ffar4*, *Scd1,* and *Scd2,* mirroring the knockdown of *Mdm2* mRNA (Fig. 3d). Furthermore, ectopic expression of MDM2 in the mature adipocytes augmented the expression of *Ffar4*, *Scd1,* and *Scd2* (Fig. 3e, Supplementary Fig. S1l). Collectively, these data render it possible that reduced levels of adipose MDM2 may regulate hepatic steatosis through impaired desaturation and secretion of the C16 and C18 NEFAs.

### Adipose MDM2 regulates adipocyte function partly through a mechanism separate from p53

The mutual interplay between the two transcription factors C/EBPα and PPARγ is indispensable for normal adipose function^32^. Interestingly, the levels of the mRNAs shown above to be decreased with lowered MDM2 levels are regulated by these two transcription factors, hinting to perturbed function of these adipocyte master regulators. In further support of this, adipocytes with lowered levels of MDM2 had decreased insulin-stimulated glucose uptake and β-adrenergic stimulated lipolysis (Supplementary Fig. S4a, b) likely caused by the lowered levels of *Glut4* and *Lipe* + *Atgl* mRNAs, respectively (Fig. 1g, h).

**Figure 4:**
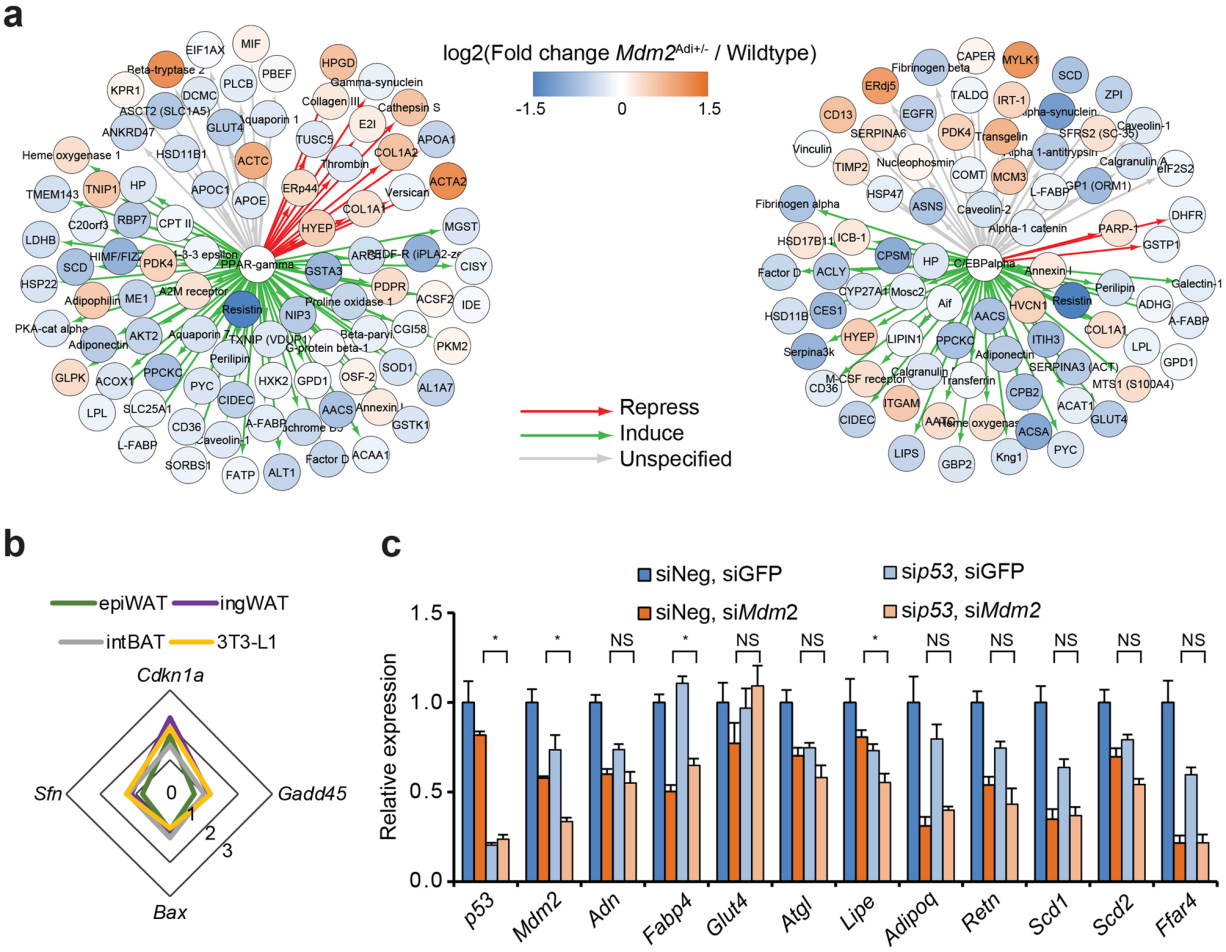
MDM2 regulation of *Ffar4* expression is at least partially independent of p53. (**a**, **b**) Data from male wildtype (N = 8) and *Mdm2*^Adi+/−^ (N = 7) mice after 15 weeks on high-fat diet. (**a**) Changes in target genes for PPARγ (left) and C/EBPα (right) in epiWAT of *Mdm2*^Adi+/−^ mice (N = 7) after 15 weeks on high-fat diet. Target genes were identified in MetaCore suite using shortest path in Build-network option and plotted in CytoScape^73^. Protein changes labeled with color with blue showing lowered levels in *Mdm2*^Adi+/−^ mice and orange higher levels. (**b**) Radar plot of relative *Sfn*, *Bax*, *Gadd45*, and *Cdkn1a* mRNA levels in epiWAT, ingWAT and intBAT from high-fat fed *Mdm2*^Adi+/−^ mice (N = 7) compared to wildtype (N = 8) after 15 weeks on high-fat diet as well as 3T3-L1 adipocytes with knockdown of *Mdm2* compared to controls as assessed by real-time qPCR. *Sfn*, 14-3-3 protein sigma; *Bax*, Apoptosis regulator BAX; *Gadd45*, Growth arrest and DNA damage-inducible protein GADD45; *Cdkn1a*, Cyclin-dependent kinase inhibitor 1. (**c**) Real-time qPCR-based quantification of *p53, Mdm2, Adn, Fabp4*, *Glut4*, *Atgl*, *Lipe*, *Adipoq*, *Retn*, *Sdc1*, *Scd2*, and *Ffar4* in 3T3-L1 adipocytes with knockdown of *Mdm2* and/or p53. For **b** and **c**, significance was tested using Student’s *t*-test, * = *p*-value < 0.05.

We then assessed the levels of known targets for PPARγ and C/EBPα in the proteomic screen of the epiWAT depots from wildtype and *Mdm2*^Adi+/−^ mice. Indeed, we found a general decrease in proteins encoded by target genes of the two transcription factors (Fig. 4a). In addition, a striking upregulation of genes reported to be repressed by PPARγ was observed (Fig. 4a). The changes in the expression of these genes could, however, not be explained by lowered levels of the transcription factors as the protein levels of PPARγ, C/EBPα and C/EBPβ (a close relative of the C/EBPα) were not altered upon *Mdm2* knockdown (Supplementary Fig. S4c) suggesting the involvement of other mechanisms.

MDM2 is best known for its ability to ubiquitinate and degrade the p53. p53 can bind and repress nuclear receptors^33^ and has recently been shown to interact with PPARγ^34^. Augmented p53 level or activity was therefore a possible candidate responsible for the alterations observed in response to lowered levels of MDM2 in vitro and in vivo. However, the p53 protein level was not augmented in epiWAT of *Mdm2*^Adi+/−^ mice (Supplementary Fig. S4d). Also, we did not observe a general activation of p53 target genes in the global proteome analysis (Supplementary Fig. S4e). Of the four classic p53 target gene mRNAs tested, only *Cdkn1a* (encoding p21) was modestly induced by decreased MDM2 expression (Fig. 4b). This was not only evident in epiWAT of *Mdm2*^Adi+/−^ mice and in vitro differentiated adipocytes but also in ingWAT and intBAT, where we observed no impact of *Mdm2* haploinsufficiency on adipocyte marker gene expression. Importantly, concomitant knockdown of *p53* did not restore the decreased adipose marker gene expression in adipocytes with lowered *Mdm2* RNA levels (Fig. 4c). Although these data do not rule out an involvement of p53, they hint that MDM2 could at least partially regulate adipose function through a mechanism distinct from p53.

### MDM2 is necessary for the nuclear localization of MORC2 and the MORC2-interacting PPARγ cofactor LIPIN1

**Figure 5:**
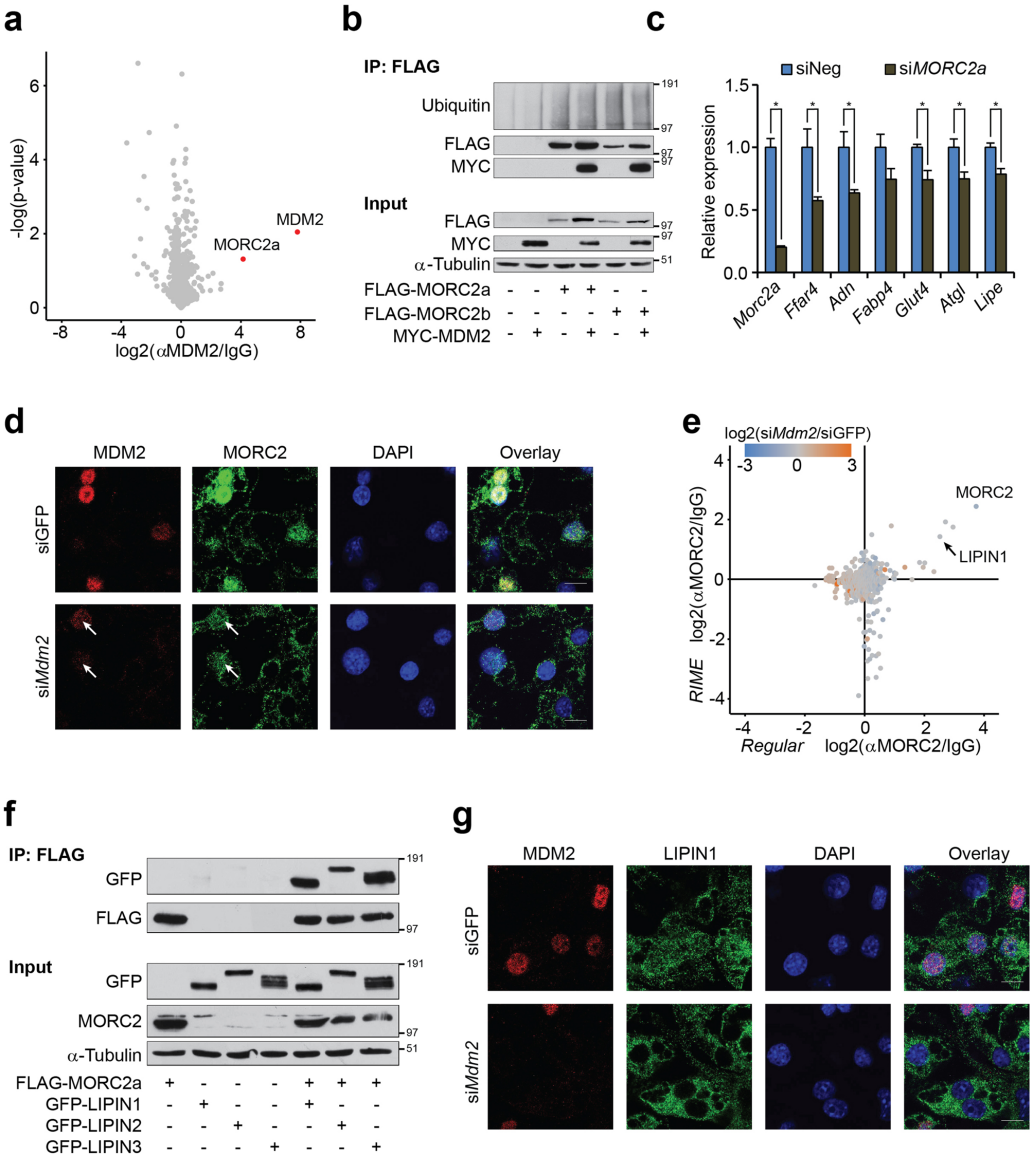
MDM2 is necessary for nuclear localization of MORC2 and LIPIN1. (**a**) Volcano plot of proteins enriched by all three monoclonal antibodies directed against MDM2 in lysates from 3T3-L1 adipocytes. Plot generated in Perseus software^70^. (**b**) Coimmunoprecipitation against FLAG in lysates from cells ectopically expressing FLAG-MORC2s and/or MYC-MDM2. Western blot analysis against Ubiquitin, FLAG and MYC. α-Tubulin was included as loading control. (**c**) Real-time qPCR-based quantification of *Morc2a*, *Ffar4*, *Adn*, *Fabp4*, *Glut4*, *Atgl*, and *Lipe* in 3T3-L1 adipocytes with knockdown of MORC2a. (**d**) Localization of MDM2 and MORC2 in 3T3-L1 adipocytes with knockdown of *Mdm2* as assessed by immunofluorescence. Nuclei were stained with DAPI. Grey scale bar corresponds to 10 µm. Images processed using ImageJ. (**e**) Scatterplot showing proteins enriched by MORC2 antibody with regular IP on the horizontal axis and RIME on the vertical axis. Colouring of dots refers to impact of *Mdm2* knockdown with blue indicated decreased binding and orange augmented. Plots generated using Perseus software^70^. (**f**) Coimmunoprecipitation against FLAG in lysates from cells ectopically expressing FLAG-MORC2a and/or GFP-LIPIN1, − 2, or − 3. Western blot analysis against FLAG, GFP, and MORC2. α-Tubulin was included as loading control. (**g**) Localization of MDM2 and LIPIN1 in 3T3-L1 adipocytes with knockdown of MDM2 as assessed by immunofluorescence. Nuclei were stained with DAPI. Grey scale bar corresponds to 10 µm. Images processed using ImageJ. For **a**, *p*-value was calculated using Student’s *t*-test. For **c**, significance was tested using Student’s *t*-test, * = *p*-value < 0.05.

As our data pointed towards additional causes for the perturbed adipocyte function in cells with lowered MDM2 levels beside p53, we set out to define the adipose MDM2 interactome. For this, we compared MDM2 co-immunoprecipitated proteins from SILAC-labeled cells by three different MDM2 antibodies in separate experiments (Supplementary Fig. S5a). Endogenous MDM2 was found to bind to the zinc finger-containing ATPase MORC2 (MORC family CW-type zinc finger protein 2) in adipocytes by all three antibodies (Fig. 5a, Supplementary Fig. S5b, Supplementary Table S2).

Although MORC2 is described as an epigenetic regulator in various settings and is mutated in the neuronal disorder Charcot-Marie-Tooth disease^35,36^, it has been shown to exert cytoplasmic function in adipocytes, where it regulates lipogenesis through ACLY (ATP-citrate synthase)^37^. We confirmed the ability of MDM2 to interact with the two murine isoforms of MORC2, namely MORC2a and MORC2b, without affecting their ubiquitination status (Fig. 5b). Both paralogues are expressed in adipocytes albeit MORC2a at higher levels compared to MORC2b (Supplementary Fig. S5c). In support of altered MORC2 function, we found perturbed expression of *AgrBP2* in adipocytes with lowered MDM2 levels both in vitro and in vivo (Supplementary Fig. S5d). *AgrBP2* has been reported to be regulated by MORC2^38,39^. Knockdown of MORC2 led to decreased expression of several adipocyte markers, and most notably *Ffar4*, supporting a role for MORC2 in mediating the effect of MDM2 (Fig. 5c).

As MORC2 has been shown to locate both to the nucleus and the cytoplasm in adipocytes, we examined its localization in adipocytes with knockdown of MDM2. Whereas MORC2 exhibited both a nuclear and a speckled cytoplasmic distribution in control cells, knockdown of MDM2 led to nuclear exclusion of MORC2 (Fig. 5d). Only a weak nuclear localization of MORC2 was seen in cells with partial knockdown of MDM2, resembling the decreased MDM2 expression observed in *Mdm2*^Adi+/−^ mice (Fig. 5d, white arrows).

Although MORC2 is known to act as a transcriptional cofactor, it has not previously been shown to bind or affect transcription factors controlling adipocyte function. We explored its interactome in the presence and absence of MDM2 using regular immunoprecipitation (Supplementary Fig. S5e). As a complementary approach, we performed a similar enrichment from crosslinked cells using RIME (Rapid immunoprecipitation mass spectrometry of endogenous proteins)^40^. The PPARγ coactivator LIPIN1 was found as one of the strongest MORC2 interaction partners and the binding of LIPIN1 to MORC2 was unaffected by lack of MDM2 (Fig. 5e, Supplementary Table S3).

LIPIN1 is the founding member of a small family of three proteins. Cytoplasmic LIPINs are involved in TG synthesis, while nuclear LIPINs partake in the coactivation of transcription factors, including PPARγ^15,41,42^. MORC2 was able to bind all LIPIN family members in co-immunoprecipitation experiments (Fig. 5f). Whereas numerous studies found a high degree of redundancy between the LIPINs, it is generally accepted that LIPIN1 is the most critical regulator of adipose biology of the three^43–45^. In support of this notion, we found a decrease in the expression of our panel of adipocyte markers upon knockdown of LIPIN1. Again, the mRNA level of *Ffar4* was the one most severely affected (Supplementary Fig. S5f.). Interestingly, knockdown of MDM2 showed that the nuclear localization of LIPIN1 in adipocytes was highly dependent on the ubiquitin ligase as well (Fig. 5g). Collectively, these data indicate that MDM2 can partially regulate adipocyte function by maintaining a nuclear pool of LIPIN1, likely via its strong interaction with MORC2.

## Discussion

Here, we show that lack of one functional allele of *Mdm2* in adipose stores leads to increased fat accumulation, glucose intolerance, and hepatic steatosis possibly related to decreased circulating levels of unsaturated lipokines due to perturbed expression of genes crucial for maintaining adipose homeostasis.

The canonical target for MDM2 is the transcription factor p53. The lipodystrophic phenotype of mice completely lacking MDM2 in adipose stores due to unrestricted p53 activity underscores the importance of their interplay in this tissue^11^. Yet, we found no overall trend for upregulated p53 activity and only a modest increase in the expression of the p53 target, *Cdkn1a*, encoding p21 in the adipose tissue of *Mdm2*^Adi+/−^ mice, arguing that the residual MDM2 was sufficient to maintain p53 activity at near normal level. Together with an inability of concomitant knockdown of p53 to fully restore adipose marker gene expression in cells with *Mdm2* knockdown, these findings prompted us to investigate if MDM2 was intertwined with other processes that could contribute to the adipose dysfunction upon MDM2 deficiency besides p53.

Our data indicate that MDM2 can facilitate nuclear sequestering of the two transcriptional cofactors MORC2 and LIPIN1 (Supplementary Fig. S5g). Knockdown of either leads to lowered expression of the same set of marker genes as *Mdm2* knockdown supporting at least a partial involvement of MORC2 and LIPIN1 in explaining the adipose phenotype of the *Mdm2*^Adi+/−^ mice. Nuclear exclusion of cofactors or transcription factors themselves is a commonly used mean to reduce gene expression. Regulation of nucleocytoplasmic shuttling of transcriptional regulators by MDM2 has to our knowledge so far only been reported for p53^46^. Shuttling of MDM2 itself is regulated by posttranslational modifications. Phosphorylation of MDM2 by AKT induces its nuclear entry^47^. Activated AKT augments transcription of PPARγ target genes at least in part due to nuclear depletion of FOXO proteins having an inhibitory effect on PPARγ^48,49^. It is, however, possible that increased nuclear import of MORC2 and LIPIN1 by MDM2 contributes to the positive impact of AKT on PPARγ. As MDM2 is known to ubiquitinate and induce degradation of FOXOs^50^, MDM2 could have multiple roles in the activation of PPARγ downstream of AKT.

Both MORC2 and LIPIN1 have previously been reported to fulfill cytoplasmic and nuclear roles. In the cytoplasm, MORC2 augments the activity of ACLY^37^ and thereby generation of acetyl-CoA, and LIPIN1 is the main producer of diacylglycerol in adipocytes^51^. Both acetyl-CoA and diacylglycerol are key intermediates in the generation of TGs. Perturbed function and/or simply increased cytoplasmic MORC2 and LIPIN1 levels due to dysfunctional nuclear import could led to augmented TG production and hence explain the obesogenic phenotype in the *Mdm2*^Adi+/−^ mice.

Besides interaction with LIPIN1, we found tight association of MORC2 with several mitochondrial proteins arguing for localization of this protein to the mitochondria as well. This was supported by its speckled localization outside the nucleus in the mature adipocytes. Hence, MORC2 may in conjunction with MDM2 play a hitherto undescribed role in mitochondrial function. Of note, compared to wildtype mice, epiWAT in *Mdm2*^Adi+/−^ mice exhibited lower levels of several proteins in the electron transport chain. Lowered mitochondrial oxidative capacity, possibly caused by MORC2 dysfunction in the epiWAT of these mice, could contribute to the obese and glucose intolerant phenotype. Yet, we failed to detect a difference in the energy consumption between the two genotypes. This could be due to the inability of indirect calorimetry to reliable measure small variations in energy expenditure, which over time could amount to altered weight gain^52–55^. In addition, the shift from being group-caged during the feeding to the single-caging for the indirect calorimetry and back again could have induced anxiety and stress amongst the mice and masked a possible difference in energy expenditure between the wildtype and *Mdm2*^Adi+/−^ mice.

The *Mdm2*^Adi+/−^ mice displayed a striking difference phenotype between their adipose depots. Whereas the expressions of several adipose markers were lowered in the epiWAT, none of those were affected in the ingWAT. The mechanism behind such differences between adipose depots remains to be elucidated. The larger metabolic adoptability of the subcutaneous depot might be able to circumvent the need for the MDM2-MORC2-LIPIN1 axis for upholding normal function.

The expression of *Ffar4*, encoding the GPR120 receptor, was consistently one the most affected genes by manipulation of MDM2 levels. GPR120 has been positioned as one of the most attractive targets for relieving complications associated with obesity, and numerous pharmaceutically interesting drugs targeting this receptor have already been developed^56^. Mice devoid of GPR120 may suffer from obesity, decreased insulin sensitivity, and fatty livers due to decreased circulating levels of palmitoleate^31^. Although discrepancies in the literature do exist on the involvement of GPR120 in the beneficial effect of omega-3 fatty acids^57,58^, the necessity for this receptor in metabolic homeostasis is a consistent finding. The *Mdm2*^Adi+/−^ mice in the current study recapitulated the phenotype of the *Ffar4*-decificent mice^31^ with a concomitantly decreased level of 16:1 palmitoleic acid, and express lower levels of *Ffar4* in their epiWAT. It is therefore conceivable that the lowered levels of *Ffar4* is at least partly responsible for the phenotype of the *Mdm2*^Adi+/−^ mice.

Collectively, our data points to an involvement of MDM2 in maintaining adipocyte homeostasis and a possible involvement in this of a hitherto unknown interplay between MDM2 and the two transcriptional cofactors, MORC2 and LIPIN1. Whether the ubiquitin/ubiquitin-like ligase activity of MDM2 is involved and needed for these functions is at the current stage unclear and awaits further investigations. It is, however, interesting that LIPIN1 has been shown to undergo both ubiquitination and sumoylation. Whereas the first leads to degradation of LIPIN1^59–61^, the latter is necessary for its nuclear localization^62^. It is intriguing to speculate that through regulation of the type of conjugate, ubiquitin or ubiquitin-like modifier, MDM2 can balance the localization and level of LIPIN1 in adipocytes.

## Methods

### Plasmids

pcDNA3-Myc-*Mdm2* was a generous gift by Dr. Jean-Christophe Marine. GFP-*LIPIN1*, GFP-*LIPIN2*, and GFP-*LIPIN3* were kindly provided by Dr. Moritz Mall. The coding region of murine *Morc2a* and *Morc2b* were amplified from a 3T3-L1 adipocyte cDNA library using the following primers: *Morc2a* forward 5’-actgctcgagatggccttcaccaattacagcagtctc-3’; *Morc2a* reverse 5’-actgctcgagtcagtcccctttggtgatgaggtcc-3’; *Morc2b* forward 5’-actgctcgagatggcgtttaccaattacagcactctc-3’ and *Morc2b* reverse 5’-actgctcgagtcagtcatcactgctgacaagatcctc-3’. Amplicons were digested with XhoI and inserted into pCMV-Tag2B. The orientation was confirmed by restriction enzyme digestion, and the constructs were sequenced.

### Animals

All animal experiments were conducted in accordance with Danish national guidelines (Amendment #1306 of November 23, 2007) approved by the Danish Animal Experiments Inspectorate, Ministry of Justice (# 2007/562–48) and in compliance with the ARRIVE guidelines. Mice were group-caged and kept under specific pathogen free conditions at 22 °C in 12 h light/dark cycle (7 AM–7 PM). The transgenic animals with ablation of *Mdm2* expression in adipose stores were generated by crossing loxP-*Mdm2* mice (kindly provided by Dr. Guillermina Lozano) on a C57Bl/6J background with *Fabp4-Cre* mice, also on a C57Bl/6J background. The mice were bred for at least 10 generations to avoid any laboratory-specific background interference.

Mice were kept on a regular chow or 45 kcal% Fat (D12451, Research Diets, New Brunswick, New Jersey) high-caloric diet for a total of 15 weeks. Mouse weight gain, food consumption, and body composition determined by magnetic resonance (MR) scanning (EchoMRI, Houston, Texas) were routinely scored. After 11 weeks on high-fat diet, mice were feed-deprived for 5 h prior to glucose tolerance tests (GTT). Fasting blood glucose was measured by tail vein bleeding using the Bayer Contour glucometer (Bayer Health Care). Mice were subsequently gavaged with 3 mg glucose/g lean mass (Fresenius Kabi). Blood glucose was measured at time points 15, 30, 60, 90, and 120 min post glucose administration. After 13 weeks on high-fat diet, mice were feed deprived 2 h prior to insulin tolerance tests (ITT). Fasting blood glucose was measured by tail vein bleeding prior to intraperitoneal injection with 0.75 mU insulin/g lean mass. Insulin (Actrapid Penfill, Novo Nordisk) was diluted in succinylated gelatin (Gelofusine, B. Braun Melsungen AG). Blood glucose was measured at time points 0, 15, 30, 60, 90, and 120 min post insulin administration. For measurements of energy expenditure after two weeks on a high-fat feed diet, O_2_ and CO_2_ gas exchange measurements were obtained for a 2-day period from each mouse using the open circuit chambers Labmaster system (TSE Systems, Bad Homborg, Germany).

Mice were killed by dislocation of the neck after 15 weeks on high-fat diet. Following decapitation, blood was isolated and allowed to clot at room temperature before centrifugation for 15 min at 1000 × *g*. Epididymal WAT, inguinal WAT, interscapular BAT, liver, and quadriceps muscle were isolated, weighed, and snap frozen in liquid nitrogen. For histology examination, parts of the adipose depots and livers were fixed in 4% paraformaldehyde in PBS over night at 4 °C.

### Fractionation of epididymal WAT

The epididymal adipose depots were excised from 8 weeks old wildtype and *Mdm2*^Adi+/−^ males, rinsed in PBS and suspended in digestion media (PBS with 1.5 unit/ml collagenase II and 10 mM CaCl_2_) and minced into small pieces. Digestion was carried out at 37 °C for 45 min with constant agitation. The suspension was passed through a 70 µm filter before centrifugation at 700x*g* for 10 min. The pellet constituted the stromal vascular fraction (SVF) and floating adipocytes the adipocyte fraction (AF).

### Cells

3T3-L1 cells were maintained in Dulbecco’s Modified Eagle Medium (DMEM) with 4.5 g/l glucose (Gibco/Thermo Fisher Scientific, Waltham, Massachusetts), 100 U/ml Penicillin–Streptomycin (Lonza, Basel, Switzerland) and 2 mM L-Glutamine (Lonza). The medium was supplement with 10% calf serum for growth and 10% fetal bovine serum for differentiation (both Sigma Aldrich).

Two days past confluence (denoted day 0), 3T3-L1 cells were induced to differentiate by supplementing the differentiation medium with 500 µM isobutylmethylxanthine (IBMX) (Merck, Kenilworth, New Jersey), 1 µM dexamethasone (Merck) and 1 µg/ml insulin (Merck). From day 2 to day 4, only insulin (1 µg/ml) was added to the differentiation medium. The medium was subsequently replaced every second day.

Knockdown was done essentially as described elsewhere^63^. In short, RNAiMAX (Thermo Fisher Scientific) was mixed with siRNA against *Mdm2* (MISSION esiRNAs (Euphoria Biotech/Merck) and/or *p53* (MISSION siRNA (Merck)) in Opti-MEM (Thermo Fisher Scientific) and aliquoted in gelatin-coated plates. Differentiating 3T3-L1 cells at day 4 were detached by trypsination, suspended in medium, spun down at 400x*g* for 5 min, and resuspended. Hereafter, cells were seeded onto esiRNA:RNAiMAX complexes. Fresh differentiation medium was added the following day and cells harvested 48 h after knockdown. For *Lipin1* and *Morc2a*, the differentiating adipocytes where transfected twice on two consecutive days with the siRNA oligos.

For lipolysis measurements, cells were rinsed twice with serum free media two days after knockdown of either GFP or MDM2. Cells were then incubated 1 h with serum free media and then stimulated with 1 µM isoproterenol (Sigma Aldrich) for 3 h. Glycerol content in the medium was analyzed with the Free Glycerol Reagent Kit (Sigma Aldrich) according to manufacturer’s instructions. For insulin stimulated glucose uptake, two days after knockdown cells were rinsed once with and incubated in serum-free media. After 1 h, the medium was replaced with Krebs–Ringer-HEPES buffer (10 mM HEPES pH 7.4, 136 mM NaCl, 4.7 mM KCl, 1.25 mM Mg_2_SO_4_) and incubation was continued for 30 min. Cells were then stimulated with insulin at the indicated concentrations for 15 min before addition of ^14^C-labeled glucose (PerkinElmer). After 15 min, glucose uptake was stopped by the addition of quench buffer (50 mM HEPES pH 7.5, 262 mM NaCl, 800 mM D-glucose), rinsed three times in ice cold PBS before lysing the cells in 1% SDS. The lysate was mixed with scintillation fluid (PerkinElmer) and radioactivity measured.

Electroporation was done as described elsewhere^64^. In short, differentiating 3T3-L1 cells at day 4 were detached by trypsination, suspended in medium, rinsed twice in PBS, resuspended in ice-cold PBS, electroporated with 100 µg of either pEGFP-C1 (Clontech, CA, USA) or pcDNA3.1-Myc-*Mdm2* and seeded in gelatin coated plates. Fresh differentiation medium was added the following day and cells were harvested 48 h after electroporation.

### Serum analysis

Triglycerides (TG) and non-esterified fatty acids (NEFAs) were measured in serum samples with Triglyceride Reagent (Sigma Aldrich) and Free Fatty Acid Quantitation kit (Sigma Aldrich), respectively, essentially according to the manufacturer’s instructions. Incubation with the lipases for the TG analyses was, however, extended to 30 min.

Adipokine levels were measured using the Proteome Profiler Mouse Adipokine Array Kit (R&D systems) according to the manufacturer’s instructions. X-ray films were quantified using ImageJ version 1.8.0 (http://rsb.info.nih.gov, National Institutes of Health, Bethesda, MD).

MUP1 levels were quantified using a MUP1 ELISA kit (Biorbyt, Cambridge, England) according to the manufacturer’s instructions. Serum samples were diluted 10 times prior to analysis. Absorbance at 450 nm read on a Victor^3^ multilabel counter (Perkin Elmer, Waltham, Massachusetts).

### Liver TG content

Liver pieces were weighed and homogenized in isopropanol, and debris pelleted by centrifugation at 16,100x*g* for 5 min. TG content was measured as described above for serum samples and normalized to the weight of the liver piece.

## Histology

Paraformaldehyde fixed tissue samples of epiWAT, ingWAT, intBAT, and liver were dehydrated and embedded in paraffin. Five-micrometer-thick sections were cut throughout the tissue and stained with hematoxylin and eosin (H & E). The area of each intact cell on each image was measured by drawing the circumference using the ImageJ open-source software, version 1.8.0. One representative micrograph from each group is presented, and tissues from four mice per group were quantified. The four animals chosen in each group were selected based on body weight and reflected the mean within each of the groups.

### Immunofluorescence microscopy

3T3-L1 adipocytes transfected with siRNA were seeded on gelatin-coated coverslips and medium replaced the following day. 2 days post transfection, cells were washed once in ice cold PBS and fixed in ice cold 4% paraformaldehyde for 10 min. Following two rinses in PBS, the cells were permeabilized (1% (w/v) BSA, 0.2% (v/v) Triton X-100 in PBS) for 10 min. at room temperature and incubated in blocking buffer (5% non-fat dry milk in PBS) for 30 min at room temperature. Coverslips were moved to a humid chamber and incubated with primary antibodies against MDM2 (4B2, a generous gift from Dr. Jean-Christophe Marine), MORC2 (SAB4301687, Merck), or LIPIN1 (5195, Cell Signaling Technology) in blocking buffer overnight in the cold room. The cells were rinsed three times in blocking buffer for 5 min each. Then, the cells were incubated with secondary antibody (Alexa488-conjugated donkey anti-Rabbit and Cy3-conjugated donkey anti-mouse (both Jackson ImmunoRearch West Grove, PA, USA)) and 0.2 ng/ml DAPI (Sigma-Aldrich) for 45 min at room temperature. The cells were rinsed three times for 5 min in blocking buffer and twice for 5 min in PBS. Coverslips were mounted with Fluorescence Mounting Medium (Dako Denmark A/S, Glostrup, Denmark).

Confocal laser scanning microscopy was performed with a Zeiss Confocal LSM 510 Meta fluorescence microscope, Carl Zeiss, Oberkochen, Germany, with a Plan Apochromat 63x/1.4 DIC oil immersion objective and a pinhole set to 1. Images were acquired at room temperature. DAPI, Alexa488 or Cy3 was excited with Lasers: Mai Tai (two photon) laser (760 nm), Argon/2 laser (488 nm) and HeNe laser (543 nm). All images were processed in ImageJ.

### RNA purification

RNA was purified using TRIreagent (Merck) according to the manufacturer’s instructions. Tissues were homogenized in TRIreagent using an Ultra-Turrax (Ika, Staufen, Germany). For adipose tissue, the homogenate was centrifuged at 16,100x*g* for 5 min, and the TRIreagent fraction without fat was transferred to another tube prior to addition of chloroform. For generation of cDNA, 1 µg of RNA was reverse transcribed using MMLV (Thermo Fisher Scientific) according to the manufacturer’s instructions. Quantitative PCR was performed in 10 μl reactions containing SYBR Green JumpStart Taq ReadyMix (Merck), 3 μl of diluted cDNA and 300 nM of each primer (Pentabase, Odense, Denmark). Reaction mixtures were preheated at 95 °C for 2 min followed by 45 cycles of melting at 95 °C for 15 s, annealing at 60 °C for 30 s, elongation at 72 °C for 45 s on the LightCycler platform (Roche). Primer specificity was checked by melting curve analysis. Sequences of primers are listed in Supplementary Table S4. *Tfiib, Tuba1a* or *Tbp* mRNA levels were used for normalization.

### Immunoprecipitation

Cells were rinsed once in ice-cold PBS before addition of lysis buffer (50 mM HEPES, pH 8.0, 150 mM NaCl, 0.5% Triton-X 100, Complete EDTA-free protease inhibitors (Roche, Basel, Switzerland), 5 mM NaF, 5 mM β-glycerophosphate, 1 mM Na_3_VO_4_). After 15 min incubation on ice, cells were scraped off and incubated 15 min on ice with short vortexing every 3–5 min. The lysate was spun down at 10,000 × *g* for 10 min and the supernatant was precleared with protein A or G beads (GE Healthcare, Little Chalfont, UK) for 60 min. For transfected 293 T cells and unlabeled 3T3-L1 adipocytes, the precleared lysate was then incubated with anti-FLAG affinity gel (Sigma-Aldrich) for 6 h and rinsed four times in lysis buffer before SDS-PAGE. For the MDM2 and MORC2 interactome experiments, 3T3-L1 adipocytes were either double SILAC-labeled with Arg6/Lys4 and Arg10/Lys8, or triple SILAC-encoded with Arg0/Lys0, Arg6/Lys4, and Arg10/Lys8^65,66^, respectively. Precleared lysates from the SILAC-labeled adipocytes were then incubated with protein G beads coupled with nonspecific IgG or antibodies directed against MDM2 (4B2, SMP14 (sc-965, Santa Cruz), or 2A10 (OP115, Merck)) or MORC2 (SAB4301687, Merck) for 6 h, combined, rinsed four times in lysis buffer and separated on a NuPAGE gel (Invitrogen) and stained with Novex Colloidal Blue (Invitrogen, Thermo Fisher Scientific). After rinsing of the gel, protein bands were excised, and subjected to in-gel digestion for subsequent LC–MS/MS analyses as described^67^.

For tissues. 2% SDS epiWAT lysates from four mice of each genotype were combined, diluted with IP buffer (150 mM NaCl, 50 mM Tris pH 8.0, 1% Triton X-100) and subjected to immunoprecipitation as described above. The MDM2 antibody SMP14 was used for the precipitation, and the 4B2 antibody was used for the western blot analysis.

### RIME (Rapid Immunoprecipitation mass spectrometry of endogenous proteins)

RIME was performed essentially as described previously^40^. In short, IgG or anti-MORC2 (SAB4301687, Merck) antibodies were preincubated with magnetic protein A Dynabeads (Thermo Fisher Scientific). SILAC-labeled 3T3-L1 adipocytes were crosslinked with 4% formaldehyde, sonicated using a Bioruptor (Diagenode, Seraing, Belgium), and lysate incubated overnight at 4 °C with antibody-bound beads. After extensive washing and combination of SILAC conditions, proteins were digested with trypsin overnight at 37 °C prior to desalting and LC–MS/MS analysis.

### Western blotting

Cells or tissues were harvested in lysis buffer (50 mM Tris HCl, pH 8,0, 500 mM NaCl, 1% NP-40, 1 mM EDTA, 0.25% NaDeoxycholate, 1.5% SDS, protease inhibitors (Complete) (Roche, Basel, Switzerland), 5 mM NaF, 5 mM β-glycerophosphate, 1 mM Na_3_VO_4_) and sonicated 2 × 5 s at 20% amplitude (Q500) (Qsonica, Newtown, Connecticut). For SDS-PAGE, 100 µg of protein was diluted 5:6 in Laemmli buffer (0.3 M Tris, pH 6.8, 12% SDS, 40% glycerol, 0.05% bromophenol blue), heated to 95 ºC for 5 min and loaded into one lane. After separation, proteins were blotted onto nitrocellulose paper (GE Healthcare, Little Chalfont, United Kingdom) using a Hoefer SemiPhor blotter. Membranes were blocked 1 h at room temperature in either 5% milk or 2% BSA in PBS/T (PBS with 0.1% Tween (Merck)). Incubation with primary antibodies was done overnight. Membranes were rinsed with PBS/T, incubated with horseradish-coupled secondary antibodies (GE Healthcare) and rinsed in PBS/T. For detection, membranes were incubated in chemiluminescence HRP substrates (Millipore, Merck, Darmstadt, Germany). Primary antibodies were directed against MDM2 (4B2, a generous gift from Dr. Jean-Christophe Marine), p53 (OP29, Calbiochem/Merck), α-Tubulin (T6199, Merck), β-Actin (sc-47778, Santa Cruz Biotechnology, Dallas, Texas), PPARγ (sc-390740, Santa Cruz Biotechnology), C/EBPα (sc-166258, Santa Cruz Biotechnology), C/EBPβ (sc-7962, Santa Cruz Biotechnology), FLAG (F7425, Merck), MYC (sc-40, Santa Cruz Biotechnology), Ubiquitin (sc-8017, Santa Cruz Biotechnology), GFP (sc-9996, Santa Cruz Biotechnology), or MORC2 (SAB4301687, Merck). Membranes for Western blotting were cut to smaller pieces prior to hybridization to reduce the amount of antibody usage and experimental cost. As full as possible length images of all Western blots are shown in Supplementary Information.

### Preparation of epiWAT extracts for SILAC-based mass spectrometry

For quantitative comparison of the adipose proteomes from wildtype and *Mdm2*^Adi+/−^ mice, epiWAT depots were homogenized and sonicated in denaturing buffer (8 M Guanidine hydrochloride (GuHCl) in 25 mM Ammonium Bicarbonate). Lysates were mixed separately 1:1 with a homogenate of epiWAT, ingWAT and intBAT depots from Lys6-fed mice. The protein mixtures were reduced with 2 mM DTT, alkylated with 11 mM chloracetamide, diluted with 25 mM ammonium bicarbonate to 2 M GuHCl and digested with LysC (1:100) overnight at room temperature. After acidification, peptides were desalted and lyophilized before high-pH fractionation as described previously^68^. Fractions were desalted before liquid chromatography mass spectrometry analyses. The analyses were performed in five replicates.

### Mass spectrometry-based proteomics

The mass spectrometric analyses were essentially performed as described earlier^68^. In short, peptide mixtures were analyzed using an Easy LC 1000 nanoflow system (Proxeon Biosystems, Thermo Fisher Scientific) connected online to a Q-Exactive HF mass spectrometer (Thermo Fisher Scientific) equipped with a nanoelectrospray ion source (Proxeon Biosystems/Thermo Fisher Scientific). For chromatographic separation, peptides were injected into a fused silica column packed in-house with 1.9 or 3 μm C_18_ beads (Reprosil) (Dr. Maisch, Ammerbuch-Entringen, Germany), applying a 120 min gradient from 8 to 64% acetonitrile in 0.5% acetic acid at a flow rate of 250 nL/min. We set up the instrument methods for the Q-Exactive HF in the data-dependent mode and the 12 most intense peaks were chosen for MS/MS fragmentation with CID activation, FTMS resolution was 60 000. Ions selected for fragmentation were dynamically excluded for 45 s, and lock mass ions *m*/*z* 391.284266, 429.0887724, 445.120024, 503.107515, 519.138815 were used for internal mass calibration^69^.

The processing of raw files from the in vivo SILAC-labeled samples was done with MaxQuant software (version 1.5.3.30, https://maxquant.net/maxquant/) with the following parameters used for searching: double SILAC with labels Lys6; maximum two missed LysC cleavages; six most intense peaks per 100 Da interval used for MS/MS peak lists; mass tolerance was 20 ppm on precursors and 0.5 Da (CID) for fragment ions. A fixed modification was carbamidomethyl (C), and variable modifications were oxidation (M), and N-term protein acetylation. Peptide, protein, and site of modifications false discovery rate (FDR) was below 1%, as assessed by the number of hits in the reverse database; minimum peptide length was seven; and a minimum of one unique peptide for protein identification was required. All unmodified and modified peptides were used for protein quantification based on both razor and unique peptides requiring a minimum ratio count of two. Only the proteins identified by at least two peptides were accepted. The raw files from the MDM2-immunoprecipitations were searched with the same parameters, except double labels were Arg6/Lys4 and Arg10/Lys8. The raw files from the MORC2-immunoprecipitations were searched with the same parameters, except SILAC labeling was Arg0/Lys0, Arg6/Lys4, and Arg10/Lys8.

The chromatographic profile and fragmentation pattern of the MUP1 peptide, DGETFQLMGLYGREPDLSSDIK, were visualized using the Xcalibur software version 4.0.27.19 (https://www.thermofisher.com/order/catalog/product/OPTON-30965#/OPTON-30965, Thermo Fisher Scientific) and the Viewer function of MaxQuant 1.5.3.30, respectively.

### Bioinformatics

Normalized, quantified proteins were imported into and plotted in Perseus v1.5.5.3 (https://maxquant.net/perseus/)^70^. Contaminants, reverse, and ‘only identified by site’ hits were sorted out. For in vivo SILAC, only proteins with a ratio in at least 2 out of 5 replicates runs were used for the Volcano plots with FDR set to 0.01 or 0.05. Proteins affiliated with the term “Blood microparticle” were mapped to the similar Volcano plots. Significantly enriched biological processes and molecular in the up- and downregulated proteins were identified using DAVID^71,72^ (https://david.ncifcrf.gov/) with the total pool of identified proteins set as the background.

To map genes regulated at the transcriptional level by p53, PPARγ and C/EBPα, the list of quantified proteins was imported into the MetaCore software (https://clarivate.com/cortellis/solutions/early-research-intelligence-solutions/, Thomson Reuters, New York, New York). We used the “Shortest path” algorithm with p53, PPARγ or C/EBPα as origin node and all proteins set as targets and filtered for “Transcriptional regulation”. Cytoscape^73^ version 2.8.0 was used for visualization of results. Cytoscape was also used to map the quantified proteins to the Wikipathway #WP295 using the Wikipathway app and coloring with continuous mapping reflecting the ratio of the identified protein.

For the immunoprecipitation of MDM2, only proteins identified in all three experiments were included in the Volcano plot. For enrichment of MORC2, only proteins identified in the regular immunoprecipitation and RIME were included in the scatter plot. Coloring was based on the average ratio between the siGFP and si*Mdm2* conditions in the two approaches.

Adope Illustrator 2020 and illustrations from the Servier Medical Art homepage (smart.servier.com) was used for the model depiction.

### Human microarray

The inverse log for the five most regulated *MDM2* probe IDs (11756046_x_at, 11753689_x_at, 11753563_x_at, 11731566_a_at, and 11753820_x_at) for the visceral depot from 5 healthy and 8 diabetic males from GEO dataset GSE78721 were averaged and normalized. These averages were plotted in a boxplot.

### Analysis of non-esterified fatty acids (NEFAs) by mass spectrometry

The analysis was carried out as previously described^74,75^. Serum samples (8 μL) were spiked with the internal standard NEFA 18:1(+ ^2^H_7_) and subjected to lipid extraction. Dried lipid extracts were dissolved in 0.75 mM ammonium formate in chloroform/methanol/2-propanol (1:2:4, v/v/v) and analyzed by negative ion mode FTMS using an Orbitrap Fusion Tribrid (Thermo Fisher Scientific) equipped with a robotic nanoflow ion source, TriVersa NanoMate (Advion Biosciences, Ithaca, NY, USA). NEFA analytes were identified and quantified using ALEX^123^ software^76^ (http://www.alex123.info/). We note here that NEFA species are identified at the lipid species-level and as such does support inference of locations of double bonds in fatty acyl chains.

## Supplementary Information

Supplementary Information 1.

Supplementary Information 2.

Supplementary Information 3.

Supplementary Information 4.

Supplementary Information 5.

## Data Availability

The mass spectrometric data generated in this study have been deposited to the ProteomeXchange Consortium (http://proteomecentral.proteomexchange.org) via the PRIDE partner repository with the data set identifier PXD020812.
