# Supplementary material for: Adipose MDM2 regulates systemic insulin sensitivity
Source: Sci Rep. 2021 Nov 8;11:21839. doi: 10.1038/s41598-021-01240-3 (PMC8575914; doi:10.1038/s41598-021-01240-3)
Supplement: Supplementary file 4 — Supplementary Information 4. [file 41598_2021_1240_MOESM4_ESM.docx]

**Supplementary table 2. Real-time qPCR primer sequences**

| Gene | Forward | Reverse |
| --- | --- | --- |
| *Acc1* | 5’-ttggcctttcacatgagatcc-3’ | 5’-gttggcgataagaaccttctc-3’ |
| *Acc2* | 5’-tggagaaggcagtccgca-3’ | 5’-ccaggatggtggctatctg-3’ |
| *Acly* | 5’-tcagccagagcttggttgtc-3’ | 5’-gaactcctccgcctgactgtgg-3’ |
| *Adgre1* | 5’-cttcccacaggcagcacag-3’ | 5’-aatgatgagaggcagcaagagg-3’ |
| *Adipoq* | 5’-gacaccaaaagggctcagg-3’ | 5’-ttaggaccaagaagacctgc-3’ |
| *Adn* | 5’-tgacagccttgagcacgac-3’ | 5’-caacgaggcattctgggata-3’ |
| *ArgBP2* | 5’-agccattaggacgactgtcg-3’ | 5’-agttgtacagacctgcgttg-3’ |
| *Atf3* | 5’-aagacagagtgcctgcagaa-3’ | 5’-gtgccacctctgcttagctc-3’ |
| *Atgl* | 5’-gggtgcgctatgtggatgg-3’ | 5’-catctctcggaggaccatgg-3’ |
| *Bax* | 5’-gctacagggtttcatccagg-3’ | 5’-gtccacgtcagcaatcatcc-3’ |
| *BmalI* | 5’-catgaaaactttgagaggtgcc-3’ | 5’-ggccaataaggtcattctggc-3’ |
| *Cd36* | 5’-caagtgcaaagaagggaaacc-3’ | 5’-tcagtgcttctatttttctagc-3’ |
| *Cd68* | 5’-cttcccacaggcagcacag-3’ | 5’-aatgatgagaggcagcaagagg-3’ |
| *Cdkn1a* | 5’-cagcgaccatgtccaatcct-3’ | 5’-cgaagagacaacggcacactt-3’ |
| *Fabp4* | 5’-acaggaaggtgaagagcatc-3’ | 5’-cctttggctcatgccctttc-3’ |
| *Fasn* | 5’-attggtggtgtggacatggtc-3’ | 5’-cccagccttccatctcctg-3’ |
| *Fabp1* | 5’-ggaaaaagtcaaggcagtcg-3’ | 5’-cgcccaatgtcatggtattgg-3’ |
| *Ffar4* | 5’-gaccaggaaattccgatttgc-3’ | 5’-cgcgatgctttcgtgatctg-3’ |
| *Fgf1* | 5’-gagcgaccagcacattcagc-3’ | 5’-ctcatttggtgtctgcgagc-3’ |
| *G6pc* | 5’-caacgtatggattccggtg-3’ | 5’-gaggctggcattgtagatgc-3’ |
| *Gadd45a* | 5’-gcagaagaccgaaaggatgg-3’ | 5’-ggtcgtcgtcttcgtcagc-3’ |
| *Lep* | 5’-gacatttcacacacgcagtcgg-3’ | 5’-ggtcattggctatctgcagc-3’ |
| *Lipe* | 5’-gacctgcttggttcaactgg-3’ | 5’-tgcctctgtccctgaatagg-3’ |
| *Mdm2* | 5’-ttctctgtgaaggaccacagg-3’ | 5’-cgcttgcaaaggatccttcag-3’ |
| *Me1* | 5’-gcagcgtcttccaaatatgg-3’ | 5’-gcaacagacgctgttccttg-3’ |
| *Mup1* | 5’-gaccctagtctgtgtccatgc-3’ | 5’-ctcttcatctcttacagtatgg-3’ |
| *p53* | 5’-tccgatggtgatggcctgg-3’ | 5’-gaaggttcccactggagtc-3’ |
| *Pck1* | 5’-gaagaaatatgacaactgttgg-3’ | 5’-catggtgcggcctttcatgc-3’ |
| *Per1* | 5’-cactctggttatgaagctcc-3’ | 5’-ctgccagctgcagtatcttc-3’ |
| *Pparg2* | 5’-acagcaaatctctgttttatgc-3’ | 5’-tgatggagaaatcaactgtgg-3’ |
| *Ppargc1a* | 5’-agccgtctctacttaagaagc-3’ | 5’-cttttgctgttgacaaatgctc-3’ |
| *Rbp4* | 5’-tccgtcttctgagcaactgg-3’ | 5’-atccagtggtcatcgtttcc-3’ |
| *Retn* | 5’-tcgtgggacattcgtgaaga-3’ | 5’-gggctgctgtccagtctatcc-3’ |
| *Scd1* | 5’-catggcgttccagaatgacg-3’ | 5’-gcttgtagtacctcctctgg-3’ |
| *Scd2* | 5’-ctgtctcttcgcgtatttgtac-3’ | 5’-cattcatacacgtcattctgg-3’ |
| *Sfn* | 5’-ccgaacggtatgaagacatgg-3’ | 5’-cgttgctcttctgctcgatgc-3’ |
| *Glut4* | 5’-ggcatcaatgctgttttctac-3’ | 5’-gctggaaccgcttccagc-3’ |
| *Tbp* | 5’-acccttcaccaatgactcctatg-3’ | 5’-atgatgactgcagcaaatcgc-3’ |
| *Tfiib* | 5’-tcgaccagccgtttggatgc-3’ | 5’-tgctgaaagttctccattcag-3’ |
| *Tnfa* | 5’-cccaaagggatgagaagttcc-3’ | 5’-acttggtggtttgctacgacg-3’ |
| *Tuba1a* | 5’-acggtcatcgatgaagttcg-3’ | 5’-tgtgcactggtcagccagc-3’ |
