# Supplementary material for: Adipose MDM2 regulates systemic insulin sensitivity
Source: Sci Rep. 2021 Nov 8;11:21839. doi: 10.1038/s41598-021-01240-3 (PMC8575914; doi:10.1038/s41598-021-01240-3)

Supplementary Information

Adipose MDM2 regulates systemic insulin sensitivity

Philip Hallenborg[1], Benjamin Anderschou Holbech Jensen[2], Even Fjære[3], Rasmus Koefoed Petersen[2], Mohammed-Samir Belmaâti[2], Sarah Søndergård Rasmussen[1], Jon Petur Gunnarsson[1], Pernille Lauritzen[1], Kenneth King Yip Cheng[4], Martin Hermansson[1], Si Brask Sonne[2], Christer S. Ejsing[1,5], Aimin Xu[6,7,8], Irina Kratchmarova[1], Marcus Krüger[9], Lise Madsen[2,3], Karsten Kristiansen[2,10,*], Blagoy Blagoev[1,*]

List of Supplementary Materials

- Supplementary Figures S1-S5
- Table legends for Supplementary Tables S1-S4 (the Tables S1-S4 are provided as separate files, not included in this document)
- Supplementary References
- As full as possible length Western blots

Supplementary Figure S1

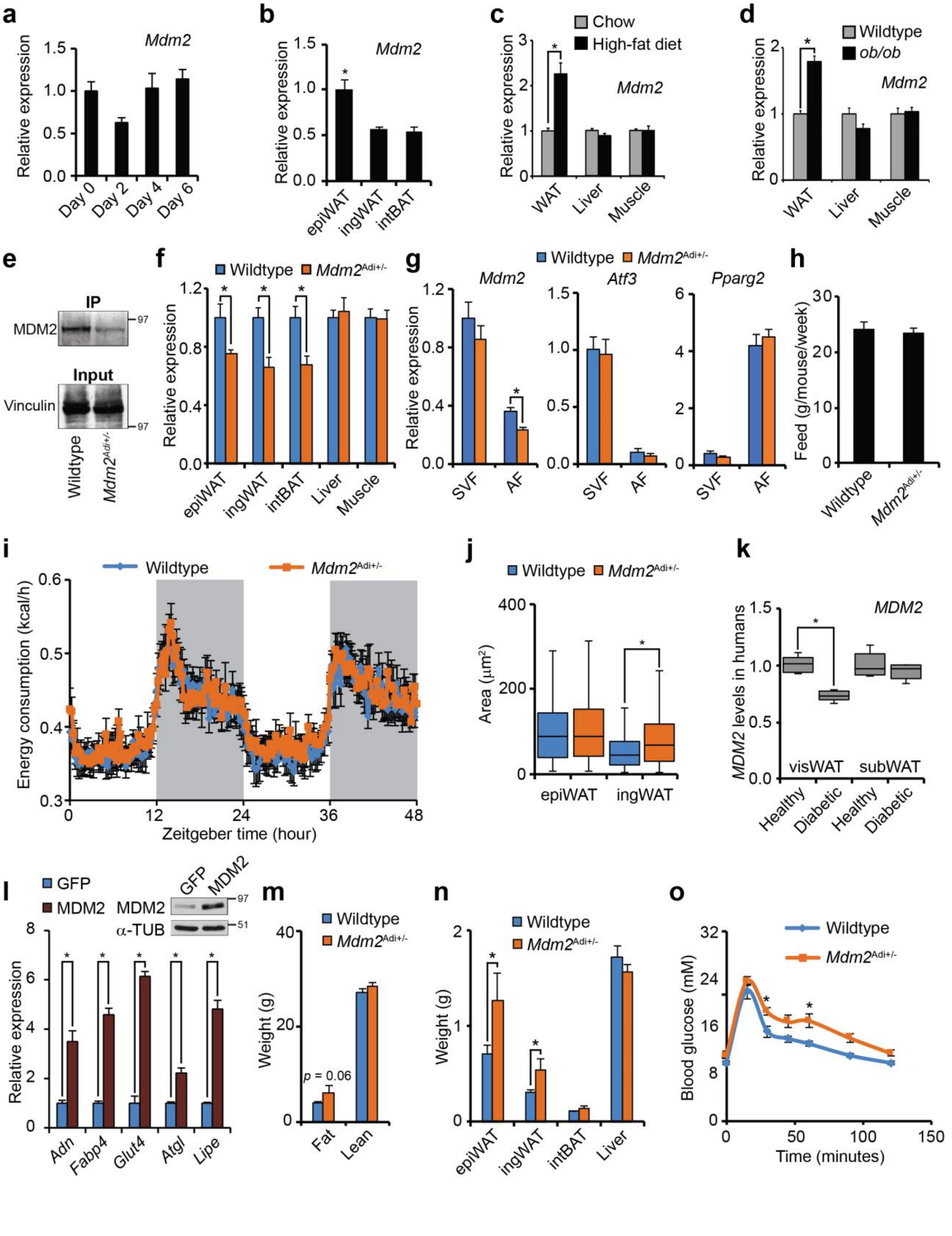

Supplementary Figure S1. Mice lacking one allele of *Mdm2* in their adipose exhibit accentuated fat accumulation and impaired insulin sensitivity when fed a high-fat diet. (a) 3T3-L1 preadipocytes were induced to undergo adipogenesis. Levels of *Mdm2* mRNA at the indicated time points were scored by real-time qPCR. (b) Expression of *Mdm2* in visceral epididymal white adipose tissue (epiWAT), subcutaneous inguinal WAT (ingWAT) and intrascapular brown adipose tissue (intBAT) as quantified by qPCR. (c) Real-time qPCR-based quantification of *Mdm2* in WAT, liver and muscle from mice fed a standard chow (N = 3) or a high-fat diet (N =3) for 15 weeks. (d) mRNA levels of *Mdm2* in white adipose tissue (WAT), liver, and muscle from wildtype (N = 5) and genetically obese, *ob/ob*, mice (N = 5). (e) Immunoprecipitation of MDM2 in pooled epiWAT lysates from wildtype (N = 4) and *Mdm2*$^{\text{Adi+/-}}$ (N = 7) mice. Levels of MDM2 in precipitate and Vinucilin in input were detected by western blot. (f) Real-time qPCR-based quantification of *Mdm2* in epiWAT, ingWAT, intBAT, liver, and muscle from wildtype (N = 8) and *Mdm2*$^{\text{Adi+/-}}$ (N = 7) mice fed a high-fat diet for 15 weeks. (g) Real-time qPCR-based quantification of *Mdm2*, *Atf3*, and *Pparg2* in stromal vascular and adipocyte fractions (SVF and AF, respectively) of fractionated epiWAT from wildtype (N = 8) and *Mdm2*$^{\text{Adi+/-}}$ (N = 8) mice. *Atf3*, Activating transcription factor 3; *Pparg2,* Peroxisome proliferator-activated receptor 2. (h) Average feed intake over 4 weeks of wildtype and *Mdm2*$^{\text{Adi+/-}}$ mice on the high-fat diet. (i) Energy expenditure of wildtype (N = 6) and *Mdm2*$^{\text{Adi+/-}}$ (N = 6) mice fed a high-fat diet for 2 weeks. (j) Boxplots of the areas of adipocytes in the epiWAT and iWAT of wildtype (N = 4) and *Mdm2*$^{\text{Adi+/-}}$ (N = 4) mice. (k) Boxplots of human *MDM2* mRNA levels in visceral and subcutaneous WAT from healthy (N = 5) and diabetic (N = 8) males. Patients were age- (>50 years) and BMI- (<30 kg/m^2) matched. (l) mRNA levels of *Adn*, *Fabp4, Glut4, Atgl,* and *Lipe* 3T3-L1 adipocytes electroporated with plasmids expressing GFP or MDM2. Insert, western blot analysis of MDM2 and α-Tubulin. (m-o) Data from wildtype (N = 9) and *Mdm2*$^{\text{Adi+/-}}$ (N = 5) mice kept on a standard chow diet. (m) Fat and lean mass of wildtype and *Mdm2*$^{\text{Adi+/-}}$ mice fed a chow diet scored by MR scanning. (n) Weight of isolated tissues. (o) Oral glucose tolerance test. For a and o, significance was tested using two-way ANOVA with Bonferroni-correction, * = *p*-value < 0.05. For b, significance was tested using two-way ANOVA with Tukey posthoc correction for multiple comparisons, * = *p*-value < 0.05. For c, d, f, g, h, j, k, l, m, and n, significance was tested using Student's *t*-test, * = *p*-value < 0.05.

Supplementary Figure S2

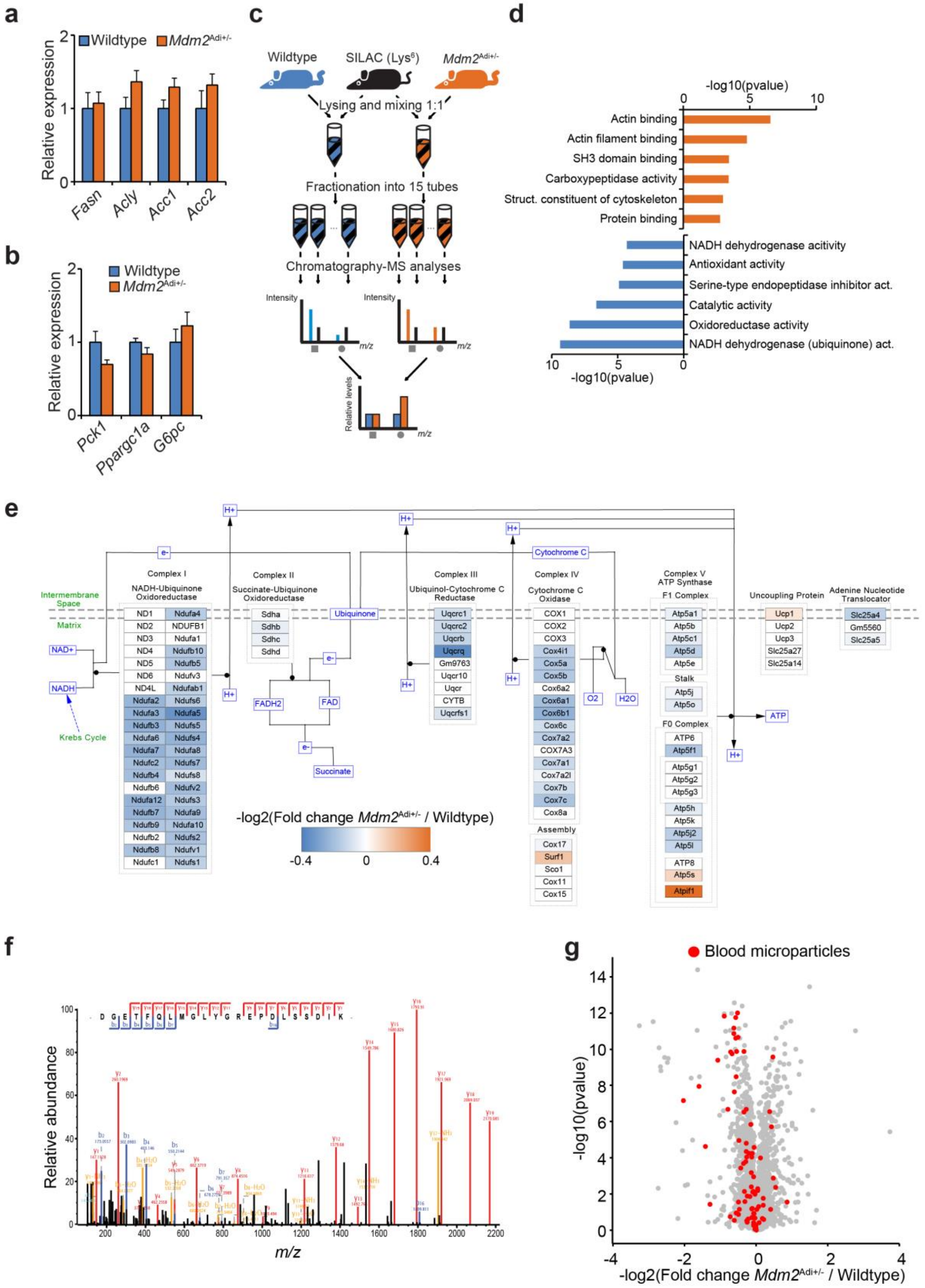

Supplementary Figure S2. High-fat fed *Mdm2*$^{Adi+/-}$ mice suffer from hepatic steatosis. (a, b) Real-time qPCR-based quantification of mRNAs encoding proteins involved in (a) *de novo* lipogenesis *Fas*, *Acly*, *Acc1*, and *Acc2* or (b) gluconeogenesis *Pck1*, *Ppargc1a*, and *G6pc* in livers from wildtype (N = 8) and *Mdm2*$^{Adi+/-}$ (N = 7) mice fed a high-fat diet for 15 weeks. *Fasn*, Fatty acid synthase; *Acly*, ATP-citrate synthase; *Acc*, Acetyl-CoA carboxylase; *Pck1*, Phosphoenolpyruvate carboxykinase; *Ppargc1a*, Peroxisome proliferator-activated receptor gamma coactivator 1-alpha; *G6pc*, Glucose-6-phosphatase. (c) Schematic generated in Adope Illustrator showing the usage of adipose tissue lysate from heavy isotope-labelled lysine-fed mice as spike-in with epiWAT extracts from wildtype and *Mdm2*$^{Adi+/-}$ mice. (d) Enriched molecular functions amongst the significantly up- or down-regulated proteins using DAVID 6.8[1,2]. (e) Mapping of quantified protein to Wikipathway #WP295 with their changes labelled with colour with blue showing lowered levels in *Mdm2*$^{Adi+/-}$ mice and orange higher levels. Plotting done in CytoScape[3]. (f) MS2 spectrum from MaxQuant[4] of the MUP1 (Major Urinary Protein 1) DGETFQLMGLYGREPDLSSDIK peptide shown in Fig 2C with b-ions in blue and y-ions in red. (g) Volcano plot of the distribution of all identified complement factors in red and proteins with the GO-term in cellular component “Blood particle” except for complement factors in black. Plotting was done using the Perseus software[5]. For a and b, significance was tested using Student’s *t*-test, * = p-value < 0.05. For d, p-value was calculated using Fisher’s Exact test with Benjamini correction. For g, p-value was calculated using Student’s t-test.

Supplementary Figure S3

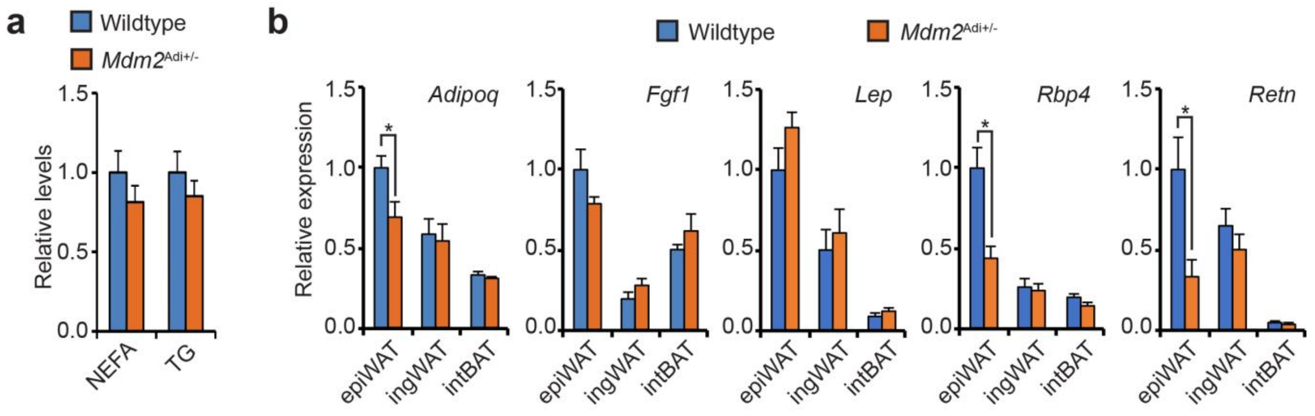

Supplementary Figure S3. High-fat fed *Mdm2*$^{Adi+/-}$ mice have disturbed adipokines secretion. (a, b) Male wildtype (N = 8) and *Mdm2*$^{Adi+/-}$ (N = 7) mice after 15 weeks on high-fat diet. (a) Serum levels of non-esterified fatty acid (NEFA) and triglyceride (TG) quantified using enzyme-based colorimetric assay. (b) Adipose mRNA levels of adipokines regulating hepatic steatosis assessed by real-time qPCR. *Adipoq*, Adiponectin, *Fgf1*, Fibroblast growth factor 1; *Lep*, Leptin; *Rbp4*, Retinol-binding protein 4; *Retn*, Resistin. For all panels, significance was tested using Student's *t*-test, * = p-value < 0.05.

Supplementary Figure S4

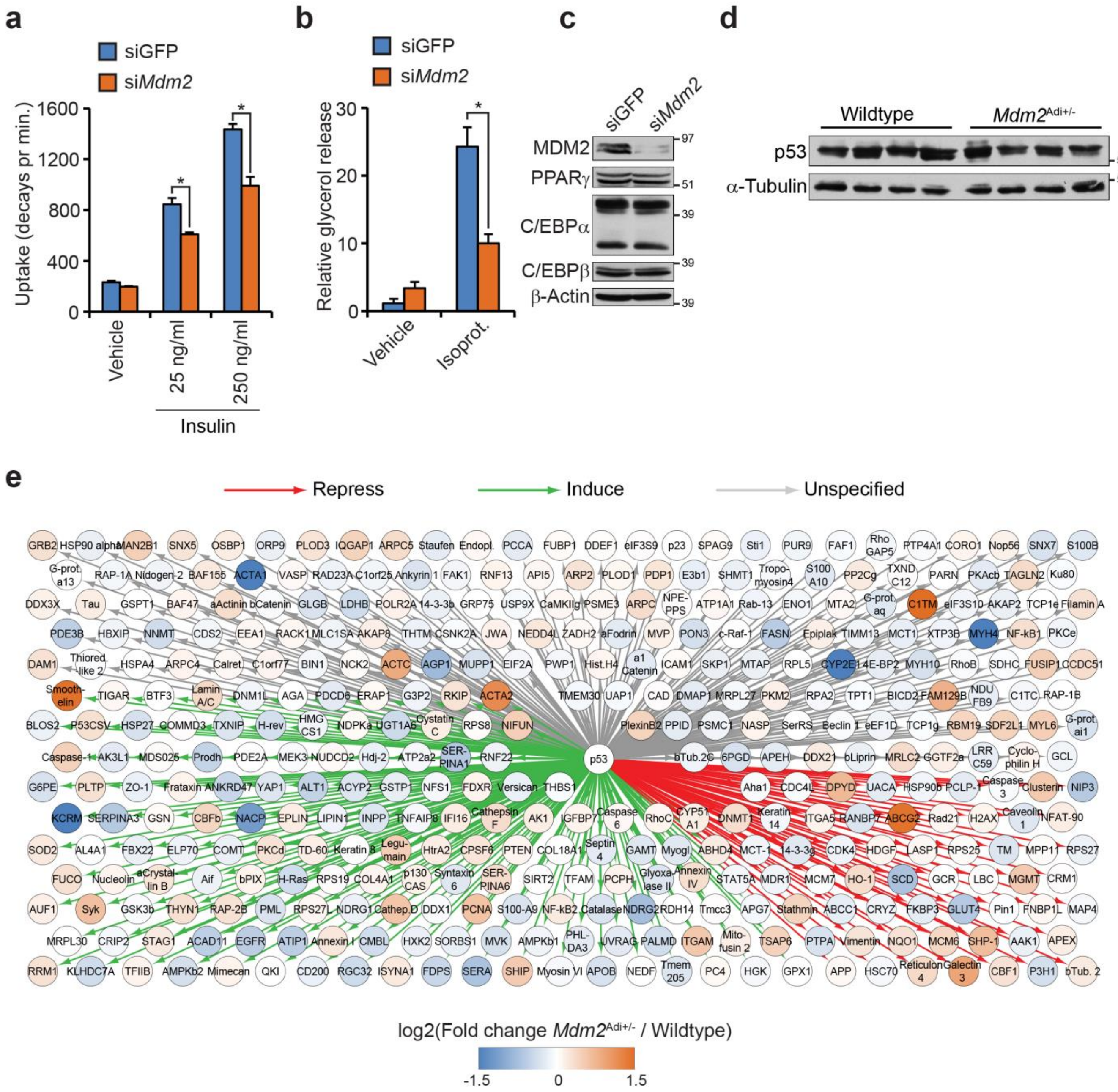

Supplementary Figure S4. MDM2 regulation of *Ffar4* expression is independent of p53. (a) Insulin stimulated glucose uptake in 3T3-L1 adipocytes with knockdown of *Mdm2*. Cells were treated with vehicle, 25 ng/ml, or 250 ng/ml of insulin, and uptake of ^{14}C-glucose was measured using scintillation. (b) Basal and isoproterenol-stimulated lipolysis in 3T3-L1 adipocytes with knockdown of *Mdm2* as scored by glycerol release using enzyme-based colorimetric assay. (c) Protein levels of MDM2, PPARγ, C/EBPα and C/EBPβ in 3T3-L1 adipocytes with knockdown of *Mdm2*. β-Actin was used as loading control. C/EBP, CCAAT/Enhancer-binding protein. (d) Protein levels of p53 in epiWAT of wildtype (N = 4) and *Mdm2*$^{Adi+/-}$ (N = 4) mice fed a high-fat diet for 15 weeks. α-Tubulin was used as a loading control. (e) Changes in target genes for p53 in epiWAT of *Mdm2*$^{Adi+/-}$ mice. Target genes were identified in MetaCore suite using shortest path in Build-network option and plotted in CytoScape [3]. Protein changes labeled with color with blue showing lowered levels in *Mdm2*$^{Adi+/-}$ mice and orange higher levels. For a and b, significance was tested using Student's *t*-test, * = *p*-value < 0.05.

Supplementary Figure S5

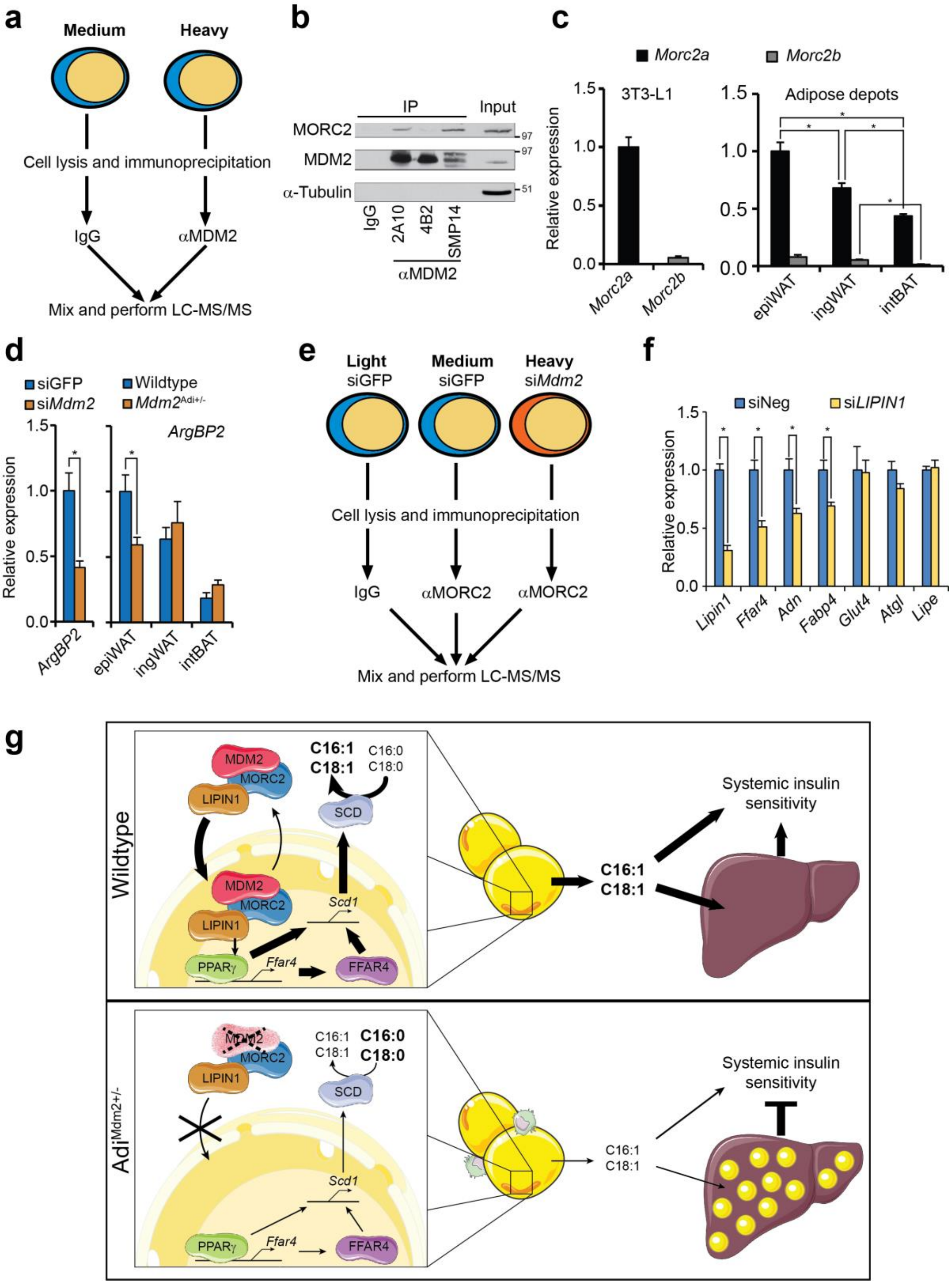

Supplementary Figure S5. MDM2 is necessary for nuclear localization of MORC2 and LIPIN1. (a) Schematic of SILAC-based quantitative proteomic setup for identifying proteins binding to MDM2. Individual IPs were performed for each of the three different monoclonal antibodies directed against MDM2 (2A10, 4B2, and SMP14). (b) Lysates of 3T3-L1 adipocytes were subjected to immunoprecipitated with IgG or monoclonal antibodies directed against MDM2. Western blot analyses of MORC2, MDM2, and α-Tubulin in immunoprecipitate and input. (c) Real-time qPCR-based quantification of *Morc2* and *Morc2b* in 3T3-L1 adipocytes (left) and epiWAT, ingWAT, and intBAT from wildtype (N = 8) and *Mdm2*$^{Adi+/-}$ (N = 7) mice fed a high-fat diet for 15 weeks (right). *Morc*, MORC family CW-type zinc finger protein. (d) Real-time qPCR-based quantification of *ArgBP2* in 3T3-L1 adipocytes with knockdown of *Mdm2* (left) and adipose depots from high-fat fed wildtype and *Mdm2*$^{Adi+/-}$ mice (right). *ArgBP2*, Arg-binding protein 2. (e) Schematic of quantitative proteomic setup for identifying proteins binding to MORC2 and the impact of *Mdm2* knockdown. (f) Real-time qPCR-based quantification of *Lpin1*, *Ffar4*, *Adn*, *Fabp4*, *Glut4*, *Atgl*, and *Lipe* in 3T3-L1 adipocytes with knockdown of LIPIN1b. (g) Putative model generated in Adope Illustrator depicting how MDM2 leads to nuclear import of MORC2 and LIPIN1, coactivation of PPARγ and thereby induction of *Ffar4*/GPR120. GPR120 is necessary for the expression of SCDs and thereby generation of palmitoleic acid which antagonizes the reduction of hepatic MUP1 expression by fatty liver. For c, significance was tested using two-way ANOVA with Tukey posthoc correction for multiple comparisons, * = p-value < 0.05. For d and f, significance was tested using Student's t-test, * = p-value < 0.05.

Supplementary Table Legends

Supplementary Table S1. Proteomic characterization of epiWAT from wildtype and *Mdm2*$^{Adi+/-}$ mice. Relative levels in 5 replicates of quantified proteins in lysates from wildtype and *Mdm2*$^{Adi+/-}$ mice. Adipose tissue lysate from heavy isotope-labelled lysine-fed mice was used as spike-in to compare across mass spectrometric analyses. Proteins were identified and quantified using MaxQuant[4].

Supplementary Table S2. MDM2 interaction partners in mature adipocytes. 3T3-L1 preadipocytes were metabolically labelled with Lys^4/Arg^6 and Lys^8/Arg^{10}, differentiated and heavy-labelled adipocytes were subjected to immunoprecipitation with three different monoclonal experiments (SMP14, 4B2, and 2A10) in separate experiments. Proteins were identified and quantified using MaxQuant[4] and relative levels of in each MDM2 IP are reported relative to IgG (Lys^4/Arg^6).

Supplementary Table S3. MORC2 interaction partners in mature adipocytes. 3T3-L1 preadipocytes were metabolically labelled with Lys^0/Arg^0, Lys^4/Arg^6 and Lys^8/Arg^{10} and differentiated. Four days after initiation of differentiation, cells were transfected with control (siGFP) (Lys^0/Arg^0, Lys^4/Arg^6) or siRNA targeting *Mdm2* (Lys^8/Arg^{10}). Adipocytes were subjected to immunoprecipitation and enriched proteins identified in mass spectrometric analyses using MaxQuant[4].

Supplementary table S4. Primer sequences. List of primer sequences used for quantitative real-time qPCR.

Supplementary References

1 Huang da, W., Sherman, B. T. & Lempicki, R. A. Systematic and integrative analysis of large gene lists using DAVID bioinformatics resources. *Nat Protoc* **4**, 44-57, doi:10.1038/nprot.2008.211 (2009).

2 Huang da, W., Sherman, B. T. & Lempicki, R. A. Bioinformatics enrichment tools: paths toward the comprehensive functional analysis of large gene lists. *Nucleic Acids Res* **37**, 1-13, doi:10.1093/nar/gkn923 (2009).

3 Smoot, M. E., Ono, K., Ruscheinski, J., Wang, P. L. & Ideker, T. Cytoscape 2.8: new features for data integration and network visualization. *Bioinformatics* **27**, 431-432, doi:10.1093/bioinformatics/btq675 (2011).

4 Cox, J. & Mann, M. MaxQuant enables high peptide identification rates, individualized p.p.b.-range mass accuracies and proteome-wide protein quantification. *Nat Biotechnol* **26**, 1367-1372, doi:10.1038/nbt.1511 (2008).

5 Tyanova, S. *et al.* The Perseus computational platform for comprehensive analysis of (prote)omics data. *Nat Methods* **13**, 731-740, doi:10.1038/nmeth.3901 (2016).

As full as possible lenght Western Blots

Figure 1h

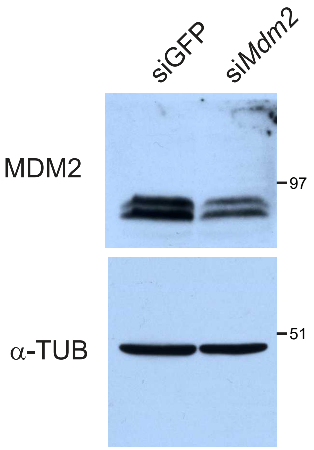

Figure 5b

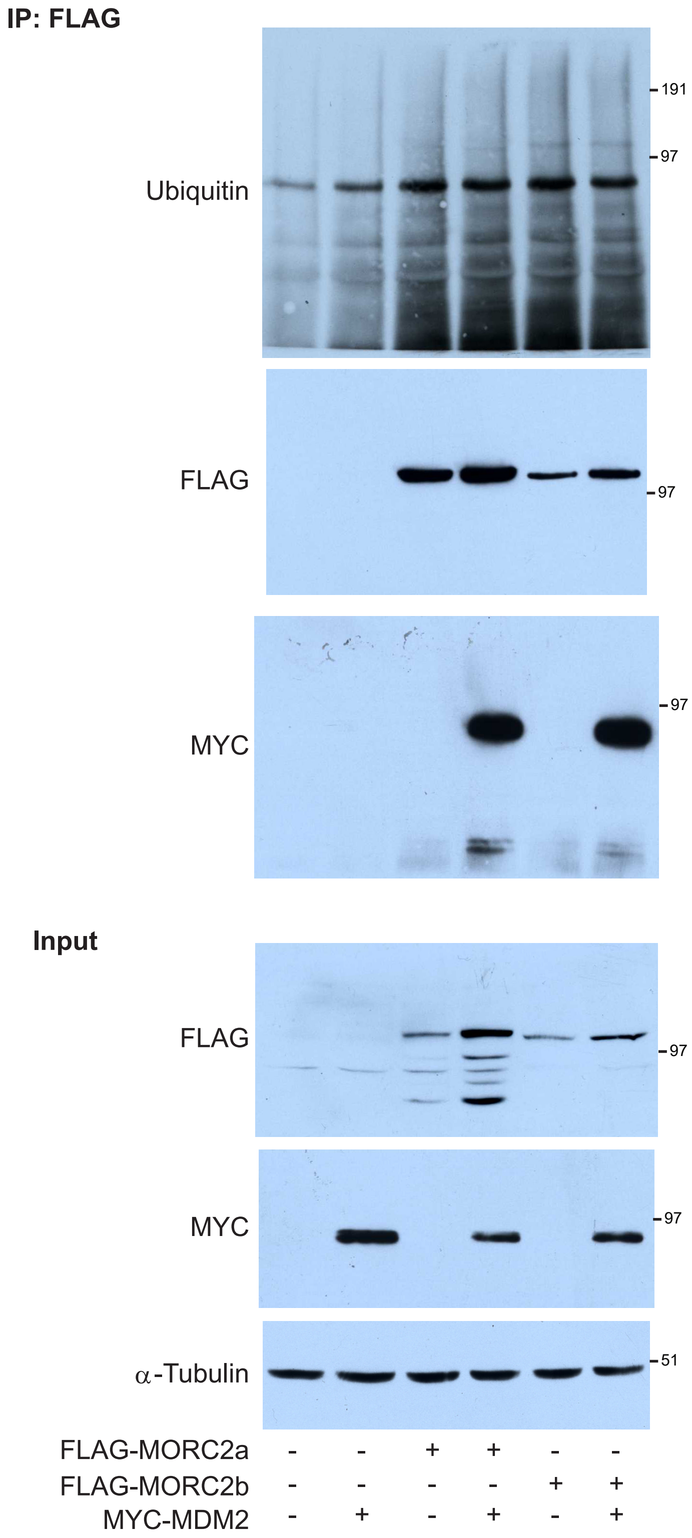

Figure 5f

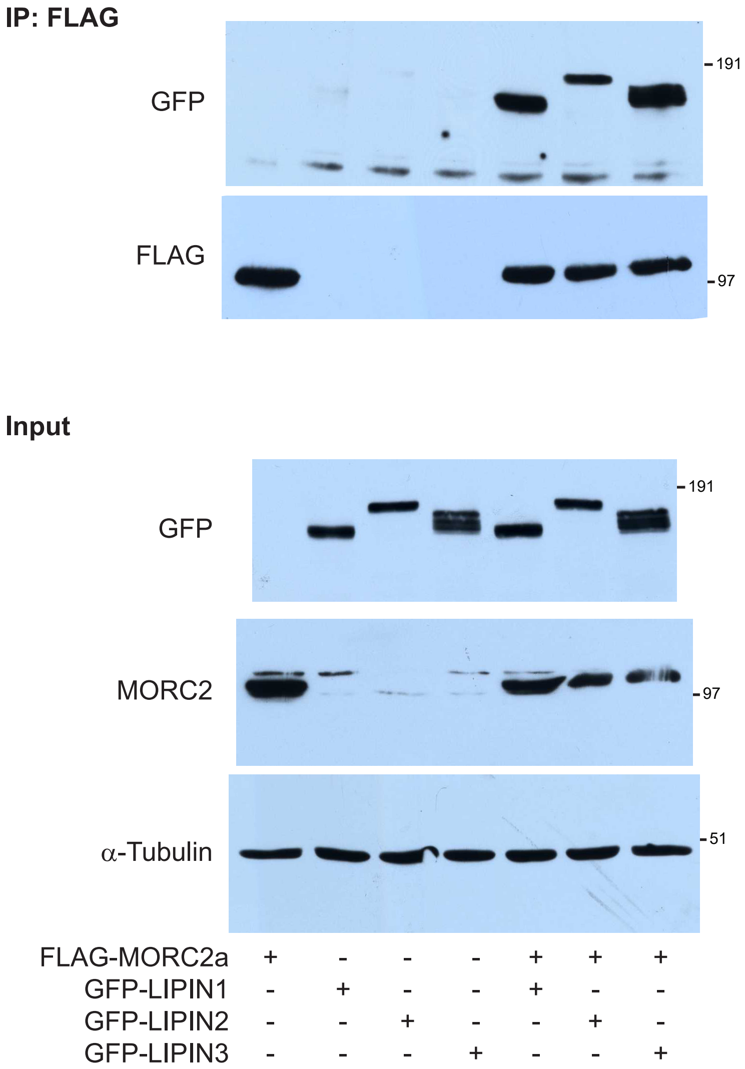

Figure S1e

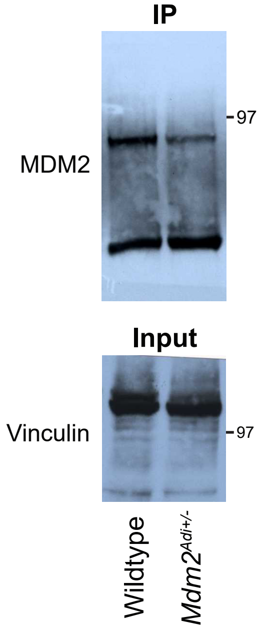

Figure S1l

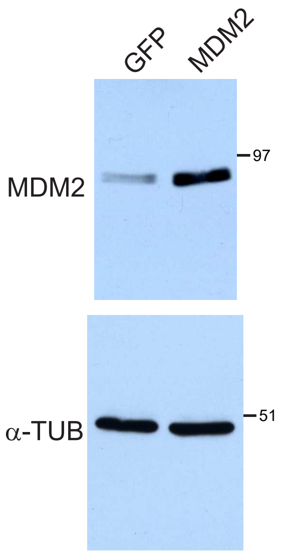

Figure S4c

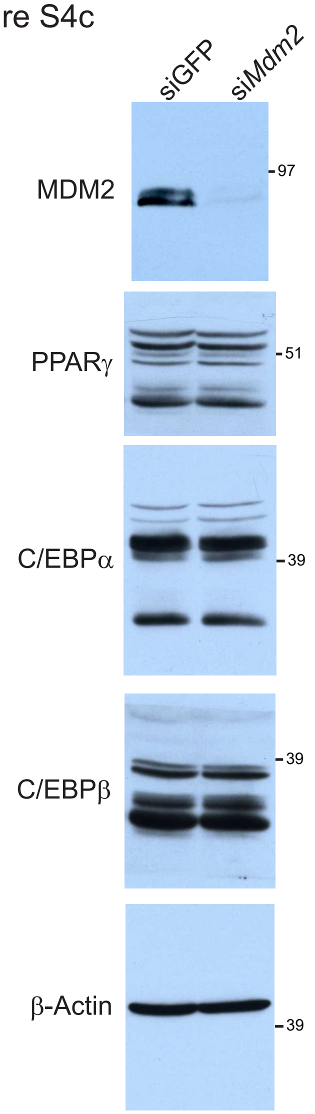

Figure S4d

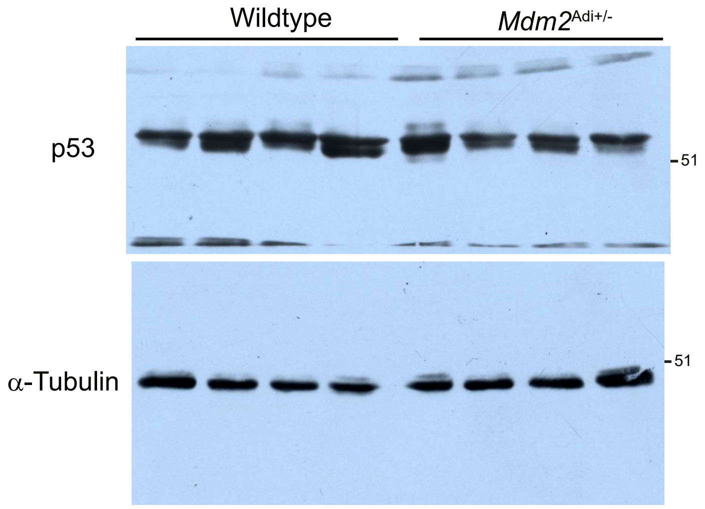

Figure S5b

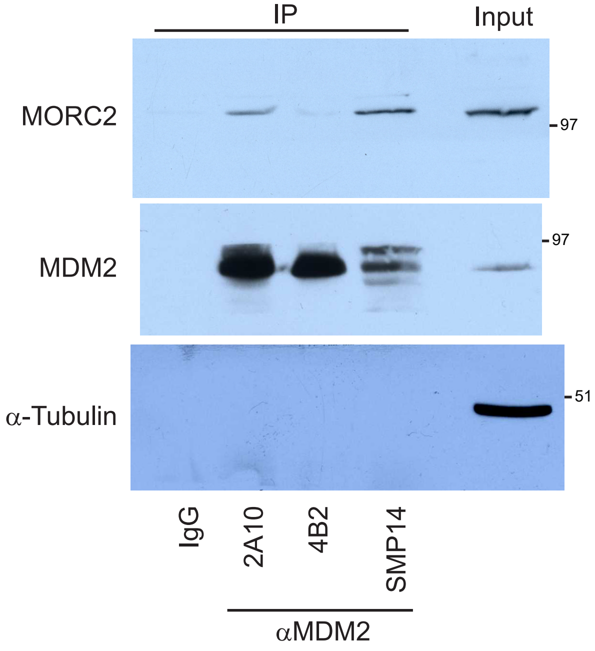

Supplement: Supplementary file 5 — Supplementary Information 5. [file 41598_2021_1240_MOESM5_ESM.pdf]
